# REGULATOR OF BULB BIOGENESIS1 (RBB1) Is Involved in Vacuole Bulb Formation in Arabidopsis

**DOI:** 10.1371/journal.pone.0125621

**Published:** 2015-04-27

**Authors:** Sang Won Han, Jose M. Alonso, Marcela Rojas-Pierce

**Affiliations:** Department of Plant and Microbial Biology, North Carolina State University, Raleigh, North Carolina, United States of America; Iowa State University, UNITED STATES

## Abstract

Vacuoles are dynamic compartments with constant fluctuations and transient structures such as trans-vacuolar strands and bulbs. Bulbs are highly dynamic spherical structures inside vacuoles that are formed by multiple layers of membranes and are continuous with the main tonoplast. We recently carried out a screen for mutants with abnormal trafficking to the vacuole or aberrant vacuole morphology. We characterized *regulator of bulb biogenesis1-1* (*rbb1-1*), a mutant in Arabidopsis that contains increased numbers of bulbs when compared to the parental control. *rbb1-1* mutants also contain fewer transvacuolar strands than the parental control, and we propose the hypothesis that the formation of transvacuolar strands and bulbs is functionally related. We propose that the bulbs may function transiently to accommodate membranes and proteins when transvacuolar strands fail to elongate. We show that RBB1 corresponds to a very large protein of unknown function that is specific to plants, is present in the cytosol, and may associate with cellular membranes. RBB1 is involved in the regulation of vacuole morphology and may be involved in the establishment or stability of trans-vacuolar strands and bulbs.

## Introduction

The lytic vacuole in vegetative cells may occupy more 90% of the cell volume. This essential organelle is highly dynamic and has critical functions in cellular homeostasis, maintenance of turgor, storage and recycling [1–3]. Vacuoles are dynamic compartments with constant membrane fluctuations and transient and highly mobile structures such as trans-vacuolar strands (TVS), sheets and bulbs [4, 5]. TVS are dynamic tubules delimited by the tonoplast that are thought to distribute cytoplasmic contents, including organelles between opposite sides of the cortical cytoskeleton [6]. In root hairs of *Hydrocharis dubia*, TVS form predomindantly along the tip growing axis and are thought to deliver cytosolic components to the growing tip [7, 8]. Many TVS sorrounding the phragmoplast were also observed in tobacco BY-2 cells during cell division [9]. The actin cytoskeleton has been localized to TVS [10], and disruption of actin filaments results in their dissasembly [11]. Similarly, microinjection of antiserum against two actin-binding proteins of the villin group disrupted the TVS in root hair cells of *H*. *dubia* [7, 12]. The current model for TVS formation is that the actomyosin system regulates TVS formation and dynamics [13, 14].

Bulbs are spherical structures of diameters between 1 and 10 μm that are highly dynamic in the lumen of the vacuole. These membrane structures are associated with the outer tonoplast membrane or transvacuolar strands and contain cytoplasmic structures, even organelles, between the folded membranes [5, 15]. Imaging by electron microscopy has indicated that bulbs are formed by multiple layers of tonoplast membranes, which explains the increased intensity of bulbs in fluorescently labeled tonoplasts [15]. Bulbs are present in LVs of cotyledon and leaf epidermis and their numbers decrease during cotyledon expansion [15], and leaf maturation [16]. Bulbs have been visualized by fluorescencence microscopy of fluorescent protein (FP) fusions with several tonoplast proteins and in multiple cell types. For example, in vacuoles of tobacco leaves, bulbs were observed when GFP-VAM3/SYP22 [17] or a GFP fusion with the phosphate transporter NPT2 [18] were transiently expressed. In Arabidopsis, bulbs have been detected in epidermis of the root elongation zone and the leaf, sepal bundle sheet, and germinating pollen of stably transformed plants expressing either GFP-AtVAM3 [5], TPK1-GFP [19, 20], TIP1;1-GFP [21], or FP fusions with other tonoplast intrinsic proteins [16, 22]. In addition, the phosphatidylinositol 3-phosphate sensor 2xFYVE-YFP also labels the bulbs [23]. These structures are not simply the result of ectopic expression of FP fusions because they were also detected in Arabidopsis WT plants by electron microscopy [15]. It was recently proposed that bulbs may form by dimerization of GFP or YFP tags when fused to tonoplast markers, and that some of these structures may be artifacts from this interaction [24]. Two types of structures were distinguished: bulbs associated with high levels of expression of GFP fusions that showed 3 or more fold increase in fluorescence intensity compared to the tonoplast, and “intra-vacuolar spherical structures” (IVSPs) present in transgenic lines in which the intensity of fluorescence was only two fold that of the tonoplast and were visualized with a non-dimerizing GFP molecule. This distinction was most dramatic in the root elongation zone where only very bright bulbs were observed, but it was more complex in other tissues, such as the cotyledon epidermis, where both types of structures existed [24].

Two hypotheses for the function of bulbs have been proposed. Bulbs may act as reservoirs of membranes for cell expansion or they may represent the first step for proteolysis of tonoplast proteins inside the vacuole [15, 19]. Only two mutants with bulb phenotypes have been identified thus far. These are *sgr2-1* and *vti11*, and in both cases these mutants show a reduced number of bulbs [23]. SGR2 encodes a protein similar to phospholipase A1, and *sgr2* mutants have an abnormal vacuole morphology with multiple small vacuole-like structures and abnormal distribution of the vacuole and the cytosol in shoot endodermal cells [25]. VTI11 encodes a SNARE (soluble N-ethylmaleimide-sensitive factor attachment protein receptor) protein that mediates membrane fusion between pre-vacuolar compartments and the vacuole [26, 27]. VTI11 is also involved in vacuole homotypic fusion, and *vti11* mutant alleles have highly fragmented vacuoles [28]. Given the highly abnormal vacuole morphology of these two mutants, it is unclear if the lack of bulbs is a direct or indirect effect of the loss of SGR2 and VTI11.

Here we describe the phenotype of *regulator of bulb biogenesis1* (*rbb1-1*), a novel mutant in Arabidopsis that displays increased numbers of bulbs in otherwise normal vacuoles. We show that *rbb1-1* mutants make fewer TVS than the parental control. We propose the hypothesis that RBB1 may function in TVS formation and that increased bulb accumulation in *rbb1-1* may result from impaired formation of TVS. In addition, we show that the differential labeling of bulbs between *rbb1-1* and the parental line does not result from abnormal trafficking to the vacuole. We also identified *RBB1* locus, which encodes a large plant-specific protein of unknown function.

## Materials and Methods

### Plant Material and Growth Conditions

The parental line carrying GFP-TIP2;1 and mCherry-HDEL [29], and the marker lines TIP1;1-YFP [16] and SYP22-RFP [30] were previously described. SALK_095160, SALK_067590 and SALK_074387 were obtained from the Arabidopsis Biological Resource Center (ABRC) and genotyped by PCR. pUBQ10::RFP-TIP1;1 was generated by Gateway cloning (Invitrogen). Briefly, the TIP1;1 CDS was amplified from Col-0 cDNA by PCR and the amplicon was introduced into pENTR-D-TOPO vector (Invitrogen). A RFP-TIP1;1 expression clone was generated by recombination with pUBN-RFP [31] using LR clonase II (Invitrogen). The promoter of the resulting construct was substituted with the full pUBQ10 promoter (1,596 bp) from pNIGEL07 (Geldner et al 2009) to generate *pUBQ10*::*RFP-TIP1;1*. The *RBB1*::*3xYpet-RBB1* and *RBB1*::*RBB1-3xYpet* constructs were generated by a Recombineering-based gene tagging system [32] using the JAtY76M24 clone and the primers listed in S1 Table. After introducing 3xYpet, the JAtY clone was shortened by homologous recombination using "delleft" and "RB-amp" primers (S1 Table). This resulted in a fragment of the chromosomal region containing 12.8 kb upstream of start codon of *RBB1* and up to 5 kb downstream from the stop codon. These constructs were transformed into Agrobacterium GV3101 and then transformed into Col-0 wild type plants via floral dip [33].

Seeds were plated on AGM (0.5X MS media and 1% sucrose) with 4 g /L GelRite. For light-grown seedlings, seeds were cold treated for 4 days in the dark to break dormancy and then incubated at 22°C under a 16 h light photoperiod. For dark-grown seedlings, seeds were cold treated in the dark and then incubated at 22°C while covered in aluminum foil.

### Chemical Stocks and Treatments

C834 (ID 6982834) was from Chembridge, Brefeldin A from Sigma and Lysotracker Red and FM4-64 from Invitrogen. All chemicals were dissolved in 100% DMSO and all treatments were carried out with 4-day old seedlings, unless specified. BFA treatment was done in liquid AGM containing 75 μM BFA and for 3 h. C834 treatment was done with 3-d-old seedlings in solid AGM-GelRite supplemented with 55μM C834 for 48 hours as described [29]. For staining, seedlings were incubated in liquid AGM containing 2 μM Lysotracker Red for 2 h or 5 μM FM4-64 for 5 min.

### Microscopy

A Zeiss LSM 710 confocal microscope was used for all imaging experiments. A 40x water objective (1.1 N.A.) was used to image embryos and roots. A 20x objective (0.8 N.A.) was used to image hypocotyls and cotyledons. The excitation/emission wavelengths during acquisition were 488 nm/492–570 nm for GFP and Ypet, 561 nm/588–696 nm for mCherry and RFP, 514 nm/588–700 nm for FM4-64, and 561 nm/566-690 nm for Lysotracker red. To count the number of TVS, seedlings were imaged using a large pinhole to generate 600 μm thick optical sections for hypocotyls and 102 μm sections for cotyledons. A 40X objective was used and image zoom was kept at 0.6. Epidermal cells within the bottom half of the hypocotyl, but excluding the root-shoot junction, were used for TVS counting in hypocotyls. Epidermal cells from the adaxial side were used for cotyledons. TVS were counted in each cell from the collected images.

### RT-PCR

Total mRNA was extracted using TRI reagent (Ambion) and treated with DNase I (NEB). cDNA was synthesized using iScript cDNA Synthesis Kit (BioRad) and was used a template for RT-PCR. Oligo sequences used for PCR are shown in S2 Table and 30 cycles were used for all reactions.

### Map-based cloning and genome sequencing

A mapping population was generated from a cross between *rbb1-1* and the L*er* wild type background, and segregating homozygous *rbb1-1* mutants were identified based on the bulb phenotype. Two Single Sequence Length Polymorphism (SSLP) markers [34] co-segregated with the *rbb1-1* phenotype and placed the mutation in Chromosome 5. For SNP detection, 12 homozygous F_3_ families from 1 backcross to the parental line were selected based on the bulb phenotype and 500 seedlings per family were used to generate DNA libraries. Samples were sequenced in an Illumina HiSeq sequencer as 100 bp reads at BGI (formerly Beijing Genomics Institute). The expected genome coverage was 35X. Data analysis was carried out at the Bioinformatics Consulting and Service Core at North Carolina State University as follows. Sequence reads were aligned to the TAIR10 genome using bowtie2 and variant calls were made using samtools mpileup. Variants were filtered for those located within TAIR10 genes and data was exported to a spreadsheet. Only SNPs located between markers RBCS-B and PINHEAD in Chromosome 5 were analyzed further. From these, mutations that were originally present in the parental line were identified from the sequencing of an unrelated mutant from the same mutagenesis screen. In addition, mutations in introns or UTRs, silent mutations and mutations unlikely to result from EMS mutagenesis were discarded.

### Western Blot

Total proteins were extracted from 7 day-old seedlings. Samples were homogenized in 50mM HEPES-KOH, pH 6.5, 5 mM EDTA, 8% sucrose, 1mM DTT and proteinase inhibitors (Complete Mini, EDTA-free, Roche Diagnostics) in a chilled mortar. The homogenates were spun at 5,000g for 10 min at 4°C and the supernatant was filtered through 3 layers of miracloth. For membrane protein fractions (P100), filtered total proteins were spun at 100,000g for 1 h at 4°C and the pellet was resuspended in buffer. Protein concentration was quantified by the Bradford assay. For immunoblot of 3xYpet-RBB1, 8 μg of proteins were loaded in a NuPAGE 3–8% Tris-Acetate gel (Life Technologies) following manufacturer instructions. The following antibodies were used: anti-GFP (Thermo MA5-15256), anti-cFBase (Agrisera AS04043), anti-H^+^ATPase (Agrisera AS07260) and anti-calreticulin [35]. Peroxidase-Conjugated Goat Anti-Mouse IgG (Thermo 32430) or peroxidase-conjugated Goat Anti-Rabbit (Thermo 32460) antibodies were used as secondary antibodies. Immunoblots were visualized by chemiluminescence with Clarity Western ECL Substrate (BioRad) using a G:BOX Chemi XRQ system (Syngene) for image capture. Signal was quantified using the GeneTools image analysis software (Syngene).

## Results

### GFP-TIP2;1 labels excess bulbs in *regulator of bulb biogenesis1*

**Fig 1:**
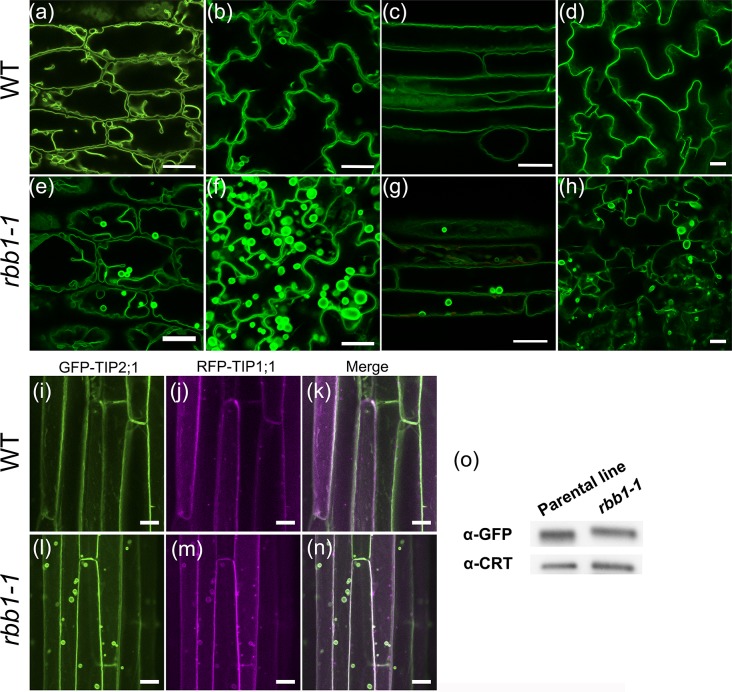
*regulator of bulb biogenesis1* contains many bulbs in many cell types. (a-h) *rbb1-1* mutants have more bulbs than the parental line. GFP-TIP2;1 localization in the parental line (WT, a-d) or *rbb1-1* (e-h). Four-day-old seedlings were imaged by confocal microscopy to visualize morphology of the vacuole in the epidermis of hypocotyl (a, e) and cotyledon (b, f), and in root epidermis and cortex (c, g). The bulb phenotype was also observed in rosette leaves of 6-week-old plants (d, h). Scale bar = 20 μm. (i-n) GFP-TIP2;1 and RFP-TIP1;1 co-localize in *rbb1-1* bulbs. The hypocotyls of 4-day-old dark-grown seedlings expressing GFP-TIP2;1 (green, i, l) and RFP-TIP1;1 (magenta, j, m) in the parental line (i-k) and *rbb1-1* mutants (l-n) are shown. Merged images (k, n) are also shown. Scale bar: 20 μm. (o) *rbb1-1* mutants do not accumulate higher levels of GFP-TIP2;1 in seedlings. Immunoblot of GFP-TIP2;1 accumulation in the parental line and *rbb1-1* using antibodies against GFP (α-GFP) and Calreticulin (α-CRT) as loading control.

We recently carried out a screen for mutants in tonoplast protein trafficking and vacuole biogenesis and characterized the function of the VTI11 SNARE protein and phosphoinositides in vacuole fusion [28]. During the course of this work, we identified a mutant in which the tonoplast protein GFP-TIP2;1 labeled many vacuolar bulbs and it was named *regulator of bulb biogenesis1-1* (*rbb1-1)*. In the parental line, the GFP-TIP2;1 protein labels the tonoplast and very few bulbs in root epidermis and cortex and the epidermis of hypocotyls, cotyledons and rosette leaves (Fig 1A–1D) [16, 28, 36]. In the *rbb1-1* mutant, GFP-TIP2;1 also labels the tonoplast, and the size and morphology of the vacuole appeared normal. However, many more bulbs were evident as bright sub-vacuolar structures in multiple tissues (Fig 1E–1H). In hypocotyls, bulbs were observed consistently in many cells in the epidermis and cortex (Fig 1E). Bulbs were also apparent in high numbers in the epidermis of cotyledons and rosette leaves of *rbb1-1* (Fig 1F and 1H). Vacuolar bulbs were detected in roots (Fig 1G), but this phenotype was variable amongst different seed stocks and was not characterized further. The variability in the root phenotype may be due to root-specific modifying factors that segregate in the population. No major defects in plant morphology were detected in *rbb1-1* homozygous plants (S1 Fig). Sequencing demonstrated that the *GFP-TIP2;1* transgene is intact in *rbb1-1* and consequently, the bulb phenotype is not related to a mutation in the GFP-TIP2;1 protein sequence.

TIP1;1-YFP is often found in vacuolar bulbs [16, 23, 37]. In order to determine if the *rbb1-1* bulbs accumulate TIP1;1 in a similar manner, we introduced a RFP-TIP1;1 fusion in the *rbb1-1* mutant background. In the parental line, RFP-TIP1;1 localized to the tonoplast, and labeled very few bulbs in roots and hypocotyls (Fig 1I–1K). RFP-TIP1;1 co-localized with GFP-TIP2;1 in the tonoplast and the bulbs when the two markers were co-expressed in *rbb1-1* (Fig 1L–1N). It was previously proposed that vacuolar bulbs are involved in the degradation of membrane proteins [19] and that bulbs may form as a response to overexpression of dimer-type FP [24]. We then tested whether the increased number of bulbs in *rbb1-1* could be caused by enhanced accumulation of GFP-TIP2;1 in *rbb1-1* by Western blot. No significant differences in GFP-TIP2;1 protein accumulation were detected between *rbb1-1* and the parental control, indicating that the increased number of bulbs in *rbb1-1* is not related to differences in protein accumulation or stability (Fig 1O and S1C Fig). These results overall indicate that, independent on the nature of the bulbs in *rbb1-1*, the *RBB1* locus is required to prevent the formation or accumulation of bulbs in the vacuole.

In order to compare *rbb1-1* bulbs with the IVSPs and bulbs previously described [24], we measured the maximum intensity values for bulb membranes in *rbb1-1*. As shown in S2 Fig, maximum intensity values for the bulbs in the parental line are 1.2- and 1.3-fold higher than the outer tonoplast in cotyledons and hypocotyls, respectively. In contrast, *rbb1-1* bulbs have 2 fold higher intensity than the tonoplast in hypocotyl, and 3.5-fold higher in cotyledons. Therefore, *rbb1-1* bulbs would fit the description of both IVSPs and bulbs [24]. Moreover, given the fact that the GFP-TIP2;1 protein levels in *rbb1-1* mutants is similar to the parental line, we conclude that the *rbb1-1* bulbs are not simply the result of increased GFP dimerization in the mutant. However, we can not discard the possibility that GFP dimerization acts as an enhancer of the *rbb1* phenotype. Given the complexity of these structures, we will refer to them here simply as bulbs.

**Fig 2:**
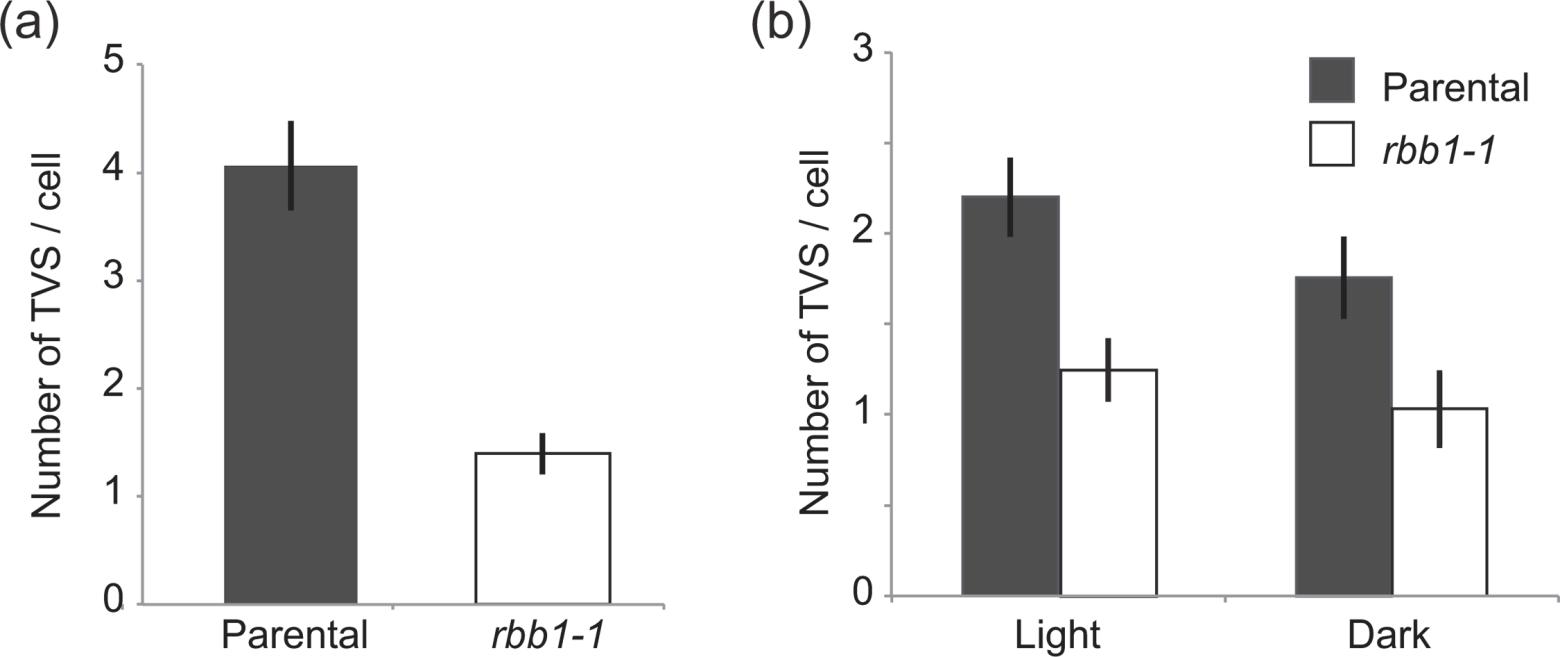
*rbb1-1* mutants have fewer transvacuolar strands. (a) The number of trans-vacuolar strands (TVS) per cell was counted in cotyledon cells from the parental line and *rbb1-1* seedlings that were grown in the light. n = 48 cells from 8 seedlings. (b) *rbb1-1* mutants have fewer TVS in hypocotyl from light- and dark-grown seedlings. Seedlings were germinated in the light or dark for 4 days and TVS were counted. n = 60 cells from 10 seedlings. Bars represent standard error. * Significantly different to the parental line in a t-test (P ≤ 0.05).

The vacuole membrane in many plant species constantly remodels with the formation of transvacuolar strands (TVS). We then hypothesized that the mechanisms involved in bulb formation and the dynamics of TVS may be related and that *RBB1* may have a role in TVS formation. In order to test this possibility, we compared the number of TVS between the parental line and *rbb1-1* mutants. In contrast to the parental line, which shows 4.06 ± 0.39 TVS per cell in cotyledon epidermis, *rbb1-1* mutants have only 1.40 ± 0.16 (Fig 2A and S3A and S3B Fig). Therefore, *rbb1-1* has ~3-fold fewer TVS than the parental line, and this result provides support to a hypothesis that RBB1 is involved in TVS formation. This phenotype was also observed in the hypocotyls of light- and dark-grown seedlings. Thus, the parental line showed an average of 2.2 ± 0.2 TVS per cell in the light and 1.75 ± 0.21 in the dark, while the *rbb1-1* mutant had 1.25 ± 0.15 and 1.03 ± 0.19, respectively (Fig 2B). This indicates that *rbb1-1* had a 40% reduction in the number of TVS per cell in hypocotyls in both light and dark-grown seedlings. These results point towards important roles of RBB1 in the remodeling of the vacuole that generates either bulbs or TVS.

### The *rbb1-1* phenotype can be detected 3 days post-germination

**Fig 3:**
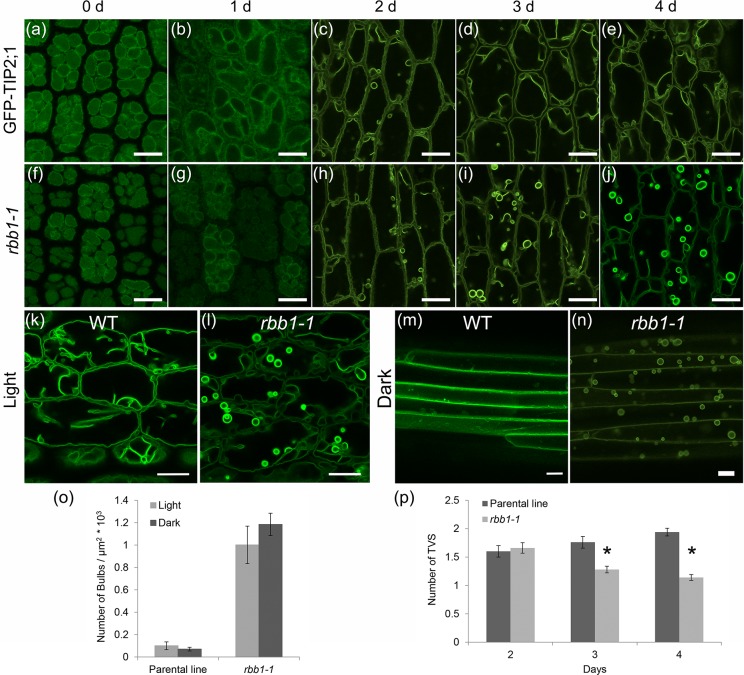
Developmental progression of the *rbb1-1* phenotype and effects from light. (a-j) The bulb phenotype is apparent in hypocotyls after 3 d of germination. Germinating seedlings from the parental line (GFP-TIP2;1, a-e) and *rbb1-1* mutants (f-j) were imaged after 0–4 days of incubation. (k-n) The bulb phenotype of *rbb1-1* is present in light- and dark-grown seedlings. GFP-TIP2;1 localization in hypocotyls was visualized in 4-d-old seedlings from the parental line (WT, k, m) or *rbb1-1* (l, n) when grown under light (k, l) or dark (m, n) conditions. n = 36 cells from 6 seedlings. All scale bars = 20 μm. (o) Dark treatment does not alter the *rbb1-1* phenotype. The number of bulbs was counted in hypocotyls from parental line and *rbb1-1* seedlings that were grown in the light or in the dark as in k-n. n = 9–15 seedlings. Bars represent standard error. (p) *rbb1-1* hypocotyls have fewer TVS after 3 d of germination. Seedlings were germinated in the light and TVS were counted after 2 days of incubation. n = 50 cells from 5 seedlings. Bars represent standard error. * Significantly different to the parental line in a t-test (P ≤ 0.01).

In order to characterize the developmental progression of the bulb phenotype in *rbb1-1*, vacuoles were visualized at different times during seed germination (Fig 3A–3J). No bulbs were observed in PSVs of imbibed seeds or 1 d after incubation at 22°C in either the parental line or *rbb1-1* (Fig 3A, 3B, 3F and 3G). Bulbs were first detected at day 2 post-germination in both the parental line and *rbb1-1* when the large lytic vacuole is already established [16]. The bulbs were very transient in the parental line and very few were observed at 3 days or later. In contrast, the number of bulbs in *rbb1-1* continued to increase at days 3 and 4. These results indicate that *RBB1* function may be important after day 3, but not during the early stages of germination.

One proposed function of the bulbs is to contribute membranes during cell expansion [15]. This prompted us to investigate whether the *rbb1-1* phenotype was affected by conditions that promoted cell expansion such as the elongation of hypocotyls in the dark. To this end, vacuoles from the hypocotyl epidermis of parental and *rbb1-1* seedlings that were grown either in the light or the dark were imaged in the microscope, and bulbs were counted. While the parental control seedlings grown in the light contain 0.10 ± 0.03 bulbs per 10^3^ μm^2^ (Fig 3K and 3O), the *rbb1-1* mutant contains 1.00 ± 0.16 (Fig 3L and 3O). Therefore, the *rbb1-1* mutant contains 10 fold more bulbs per area in hypocotyls when compared to the parental control grown under normal light conditions. Next we analyzed the phenotype of *rbb1-1* mutants in the dark. In contrast to the dark-grown parental control, which contains 0.07 ± 0.01 bulbs per 10^3^ μm^2^ (Fig 3M and 3O), the dark-grown *rbb1-1* mutant showed 1.18 ± 0.09 bulbs per 10^3^ μm^2^ (Fig 3N and 3O), or a 16-fold increase. Thus, the number of bulbs in *rbb1-1* was higher than the parental under both light treatments, but light availability did not significantly alter the *rbb1-1* phenotype. We then tested whether the increased number of bulbs would enhance the elongation rate of dark-grown *rbb1-1* seedlings, but no differences in hypocotyl length were detected between the mutant and the parental control at 3 or 5 d (S3C Fig). Furthermore, cell elongation was not affected, as we could not detect significant differences in cell size between the wild type and *rbb1-1* hypocotyls when grown in the dark (S3D Fig).

Finally, if the *RBB1* is responsible for the bulb and TVS phenotypes of *rbb1-1*, we expected a similar developmental progression, which can be tested by quantification of TVS during germination. TVS could not clearly be detected before day 2, and no significant difference was detected at day 2 between wild type and *rbb1-1* hypocotyls when grown in the light (Fig 3P) However, the number of TVS was reduced by 28% in *rbb1-1* when compared to the wild type. This reduction was even greater (41%) at day 4. Therefore, the appearance of the bulb phenotype at days 3 and 4 correlates well with the reduction of TVS numbers in *rbb1-1*.

### GFP-TIP2;1 trafficking to the vacuole is not altered in *rbb1-1*

**Fig 4:**
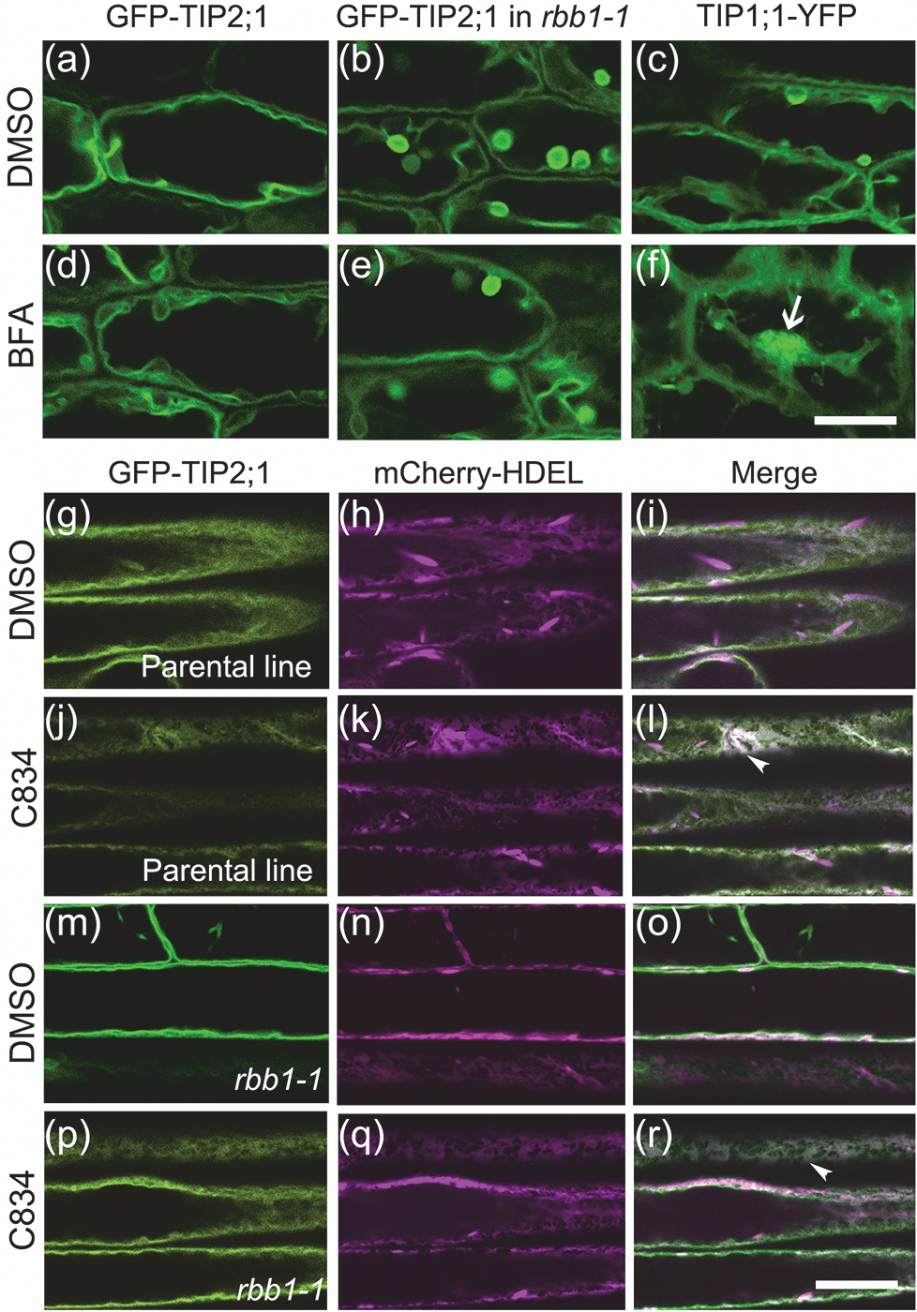
GFP-TIP2;1 trafficking to the vacuole is similar between *rbb1-1* and the parental line. (a-f) BFA inhibits trafficking of TIP1;1YFP but not GFP-TIP2;1 in the parental line or *rbb1-1*. Four-day-old seedlings from the parental (GFP-TIP2;1, a, d), *rbb1-1* (b, e) or TIP1;1-YFP (c, f) were exposed to 0.1% DMSO (control, a, b, c) or 75 μM BFA (d, e, f) for 3 hours and hypocotyl cells were imaged. BFA compartments are indicated with arrows. Scale: 20 μm. (g-r) C834 inhibits GFP-TIP2;1 trafficking in the parental line and *rbb1-1*. Three-day-old seedlings from the parental line and *rbb1-1* were exposed to 0.5% DMSO (g-i, m-o) or 55 μM C834 (j-l, p-r) for 48 h, and root cells were imaged in the microscope. Signal from GFP-TIP2;1 (green, g, j, m, p), the ER marker mCherry-HDEL (magenta, h, k, n, p) or the merged image is shown (i, l, o, r). Co-localization of GFP with mCherry-HDEL at the ER is indicated with arrowheads. Scale bar: 20 μm.

The increased number of bulbs in *rbb1-1* could be the result of abnormal GFP-TIP2;1 protein trafficking to the vacuole. To test this hypothesis, two inhibitors for tonoplast protein trafficking were used. Brefeldin A (BFA) inhibits a Golgi-dependent pathway for tonoplast proteins in hypocotyls and C834 inhibits a Golgi-independent traffic of GFP-TIP2;1 to the tonoplast [29]. We treated the GFP-TIP2;1 (parental control), the TIP1;1-YFP line and the *rbb1-1* mutant with BFA and visualized each fusion protein in hypocotyls. The fusion proteins localized to the tonoplast (GFP-TIP2;1) or tonoplast and bulbs (*rbb1-1* and TIP1;1-YFP) in the DMSO control as expected (Fig 4A–4C). In BFA-treated cells, the GFP-TIP2;1 localization was unchanged in the parental control (Fig 4D) or *rbb1-1* (Fig 4E), indicating that the trafficking of this protein was still BFA-insensitive. In contrast and consistent with previous reports [29], the TIP1;1-YFP marker line displayed large and bright aggregates in the presence of BFA (Fig 4F). Next we tested sensitivity to C834. GFP-TIP2;1 localized to the tonoplast and did not co-localize with the ER marker mCherry-HDEL in neither the parental line or *rbb1-1* (Fig 4G–4I and 4M–4O). In contrast, GFP-TIP2;1 co-localized with the ER marker in C834-treated roots of the parental line (Fig 4J–4L) and *rbb1-1* (Fig 4P–4R). Therefore, we conclude that GFP-TIP2;1 traffics in a similar pathway in *rbb1-1* and the parental line, at least early in the secretory pathway, and that the bulb-localization of GFP-TIP2;1 in *rbb1-1* does not appear to be the result of altered trafficking to the vacuole.

### The *RBB1* locus corresponds to *At5g40450*

**Fig 5:**
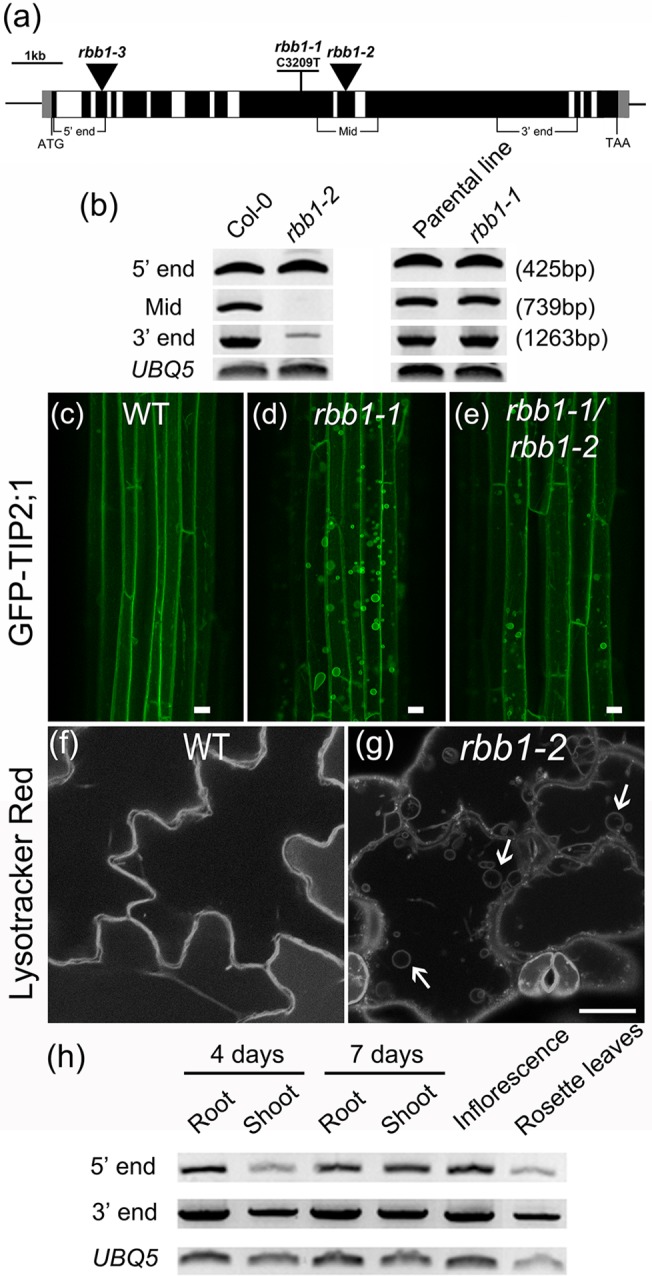
The *RBB1* locus corresponds to *At5g40450*. (a) Schematic structure of the *RBB1* locus indicating the positions of *rbb1* alleles. Exons are indicated as black boxes and introns are indicated as white boxes. 5' and 3' UTRs are indicated in gray. Triangles indicate T-DNA insertions of SALK lines. The position of three diagnostic RT-PCR amplicons at the 5’ end, middle and 3’ end are shown. (b) Accumulation of *RBB1* transcripts as determined by RT-PCR with gene specific primers in seedlings from Col-0 (WT), *rbb1-2*, the GFP-TIP2;1 parental line and *rbb1-1*. Three pairs of gene specific primers were used to detect the transcript at the 5' end, middle, and 3' end of the *RBB1* gene as indicated in (a). Numbers represent amplicon size. (c-e) Lack of genetic complementation between *rbb1-1* and *rbb1-2*. Hypocotyls of four-day-old dark-grown seedlings from the parental control (WT, c), *rbb1-1* (d) and the F_1_ progeny from a cross between *rbb1-1* and *rbb1-2* (*rbb1-1/rbb1-2* double heterozygotes, e) were imaged by confocal microscopy. Scale bar: 20μm. (f-g) *rbb1-2* contains bulbs in a similar manner as *rbb1-1*. Four-day-old seedlings were stained with 2 μM Lysotracker Red for 2 hours to label the vacuole in Col-0 (WT) and *rbb1-2*. Bulbs are indicated with arrows. Scale: 20 μm. (h) *RBB1* is expressed in many developmental stages. The accumulation of *RBB1* transcripts was determined by RT-PCR with gene specific primers in various tissues of 4 and 7-day old Col-0 (WT) plants. Two pairs of gene specific primers detected the 5' end and 3' end of *RBB1* gene as indicated in (a).

The bulb phenotype of *rbb1-1* segregated as 25 out of 96 (1/3.84) in a backcross to the GFP-TIP2;1 parental line, suggesting that the *rbb1-1* mutation is recessive. In order to identify the *RBB1* locus, we used a combination of map-based cloning and whole genome sequencing. The *RBB1* gene mapped to a region of 2.2Mb in Chromosome 5 between markers RBCS-B and PINHEAD. Using Illumina whole-genome sequencing, we identified nucleotide variants in the *rbb1-1* mutant genome between these two markers. From those, only two SNPs located within gene coding sequences and corresponding to EMS-type non-silent mutations were detected, in *At5g38540* and *At5g40450*. In order to determine which gene corresponded to the *RBB1* locus, we identified Salk T-DNA insertion alleles [38] for both candidate genes. Only one T-DNA line, SALK_095160, for *At5g38540* was available, but no homozygote plants could be recovered. Heterozygous plants were crossed to *rbb1-1* and the vacuole phenotype of F_1_ seedlings grown in the dark was analyzed. Out of 27 F_1_ seedlings, none showed the *rbb1-1* bulb phenotype indicating that *RBB1* was unlikely to correspond to *At5g38540*. We obtained two SALK T-DNA alleles for *At5g40450* (Fig 5A). However, one of the T-DNA lines, SALK_067590, had a complex transgene insertion and could not be used. We used RT-PCR to determine if *At5g40450* was expressed in SALK_074387. The expression of the full length *At5g40450* was disrupted in SALK_074387 (Fig 5B), and therefore, this was a good mutant allele to test for the bulb phenotype. However, it is unclear if this allele is a complete loss of function allele because partial transcripts can be detected by RT-PCR. We named this allele *rbb1-2*.

To determine if *RBB1* is *At5g40450*, a complementation test between *rbb1-1* and *rbb1-2* was carried out. F_1_ seedlings from a cross between *rbb1-1* and homozygous *rbb1-2* were incubated in the dark and analyzed by fluorescence microscopy for the bulb phenotype. As shown in Fig 5E, the double heterozygote F_1_ plants display the *rbb1* phenotype and indicate the lack of complementation between the two mutant alleles. We were unable to confirm this result in the F_2_ population as the GFP-TIP2;1 transgene was consistently silenced in the segregating population whenever the SALK insertion was present. This result is consistent with highly silenced transgenes in SALK insertion lines [39]. To confirm the identity of *RBB1*, we visualized the vacuole morphology of *rbb1-2* mutants with Lysotracker Red, a fluorescent dye that stains acidic organelles including plant vacuoles [40] and the tonoplast membrane [41, 42] (S4 Fig). This experiment could only be carried out in cotyledons from light-grown seedlings as penetration of fluorescent dyes in hypocotyls is very poor. Similar to the reduced number of bulbs in the GFP-TIP2;1 line, bulbs were rarely detected in wild type Col-0 cotyledons (Fig 5F and S1 Movie). On the contrary, the *rbb1-2* mutant allele showed a similar bulb phenotype as the *rbb1-1* mutant with 4–5 vacuolar bulbs per cell in these cells (Fig 5G and S2 Movie). This phenotype also segregated as ~1/4 and therefore *rbb1-2* is likely to be recessive. These results confirmed that the *RBB1* locus corresponds to *At5g40450* and that the bulb phenotype may be detected in the absence of the GFP-TIP2;1 transgene.

*At5g40450* encodes a large protein of 2,890 amino acids. The mutation in *rbb1-1* is a C/T nucleotide substitution that results in an A1070V substitution in the predicted protein sequence. BLASTp analysis in NCBI identified only two proteins with protein similarity covering at least 40% of the RBB1 protein sequence, CARUB_v10003962mg from *Capsella rubella* and EUTSA_v10027617mg from *Eutrema salsugineum*. All other hits (~40) show high similarity to a 75 amino acid region at the C terminus of RBB1. Remarkably, no significant similarity was found outside of plant taxa using BLASTp, which indicates that RBB1 is a plant-specific protein. In addition, no specific domain hits were detected using the Conserved Domain Database search function in NCBI [43], but regions of similarity to three multi-domain structures were detected. Only two proteins were identified with significant similarity to RBB1 in *C*. *rubella* and *E*. *salsugineum*. RBB1 belongs to the putative Plant Model Organism Orthologous Group APK_ORTHOMCL5144, which includes genes from Rice, Poplar, Sorghum, Maize, and Brachypodium (http://rice.plantbiology.msu.edu/cgi-bin/ortholog_group_apk.pl). This group is also supported by gene family clustering of orthologous genes in Phytozome (www.phytozome.net) [44]. The putative orthologs also encode very large proteins (up to 3,715 aa). One of the putative orthologs, *Ricinus communis* gene 29917.t000066, contains a 32 amino acid region that is 32% identical to the microtubule-associated protein futsch in *Drosophila melanogaster*. These results overall indicate that RBB1 is a novel plant-specific protein with unknown molecular function.

In order to determine the spatial and temporal expression of *RBB1*, the accumulation of *RBB1* transcripts was analyzed by RT-PCR in Col-0 wild type plants. *RBB1* accumulated at similar levels in 4- and 7-day-old light-grown seedlings, and in rosette leaves and inflorescences (Fig 5H). These results are consistent with publicly available databases for *At5g40450*. According to Arabidopsis eFP browser [45], *RBB1* is expressed in leaves and roots of both seedlings and mature plants, flowers and all stages of embryo development. Seedlings treated for 12 h with ABA, heat, cold and osmotic stress have decreased levels of *RBB1* transcripts [45, 46]. In contrast, 3 h treatment with photosystem II inhibitor N-octyl-3-nitro-2,4,6-trihydroxybenzamide (PNO8) or the brassinosteroid biosynthesis inhibitor brassinazole 220 resulted in a 2-fold induction of *RBB1* [47]. These results indicate that *RBB1* may have important functions in multiple developmental stages. The *RBB1* transcript accumulation was also analyzed in the parental line and *rbb1-1*, but no differences in the expression level were detected (Fig 5B). Therefore, the *rbb1-1* mutation did not affect the expression of *RBB1* at the transcriptional level.

### RBB1 is a cytosolic protein

**Fig 6:**
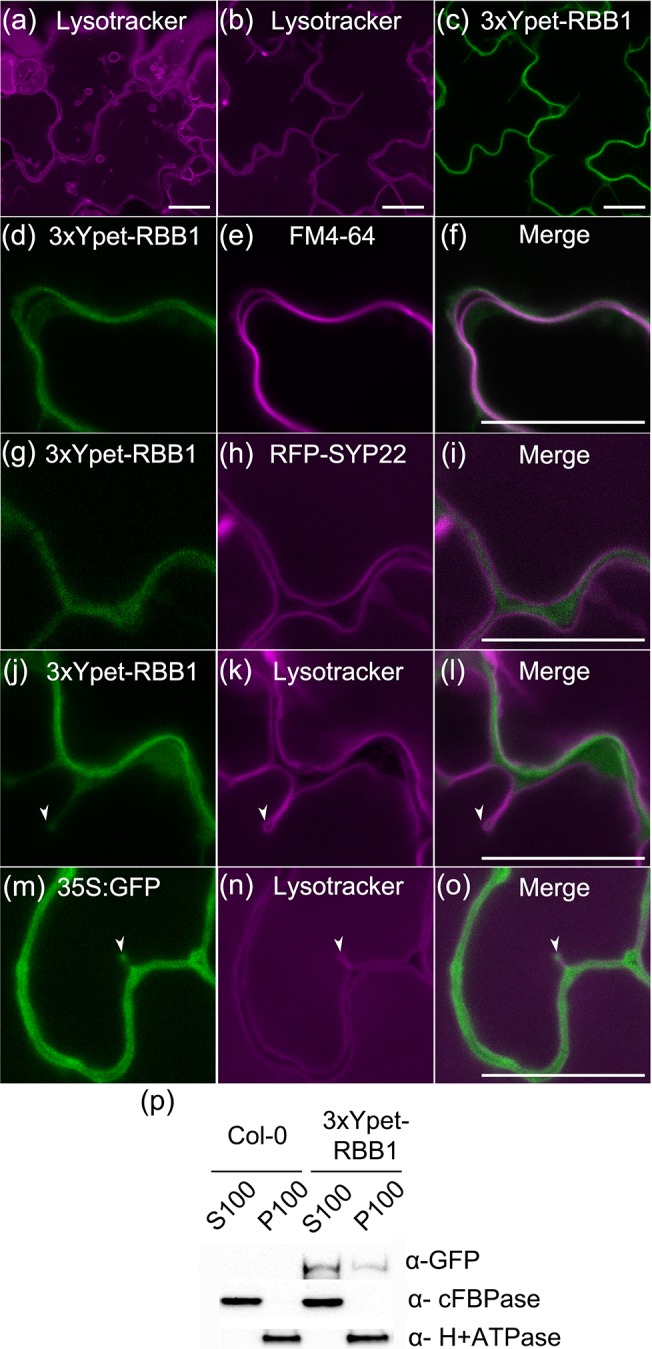
3xYpet-RBB1 is a cytoplasmic protein that associates with the tonoplast. (a-c) A 3xYpet-RBB1 fusion complements the bulb phenotype of *rbb1-2*. Four-day-old seedlings from *rbb1-2* (a) or *rbb1-2* 3xYpet-RBB1 (b, c) were stained with Lysotracker Red (magenta, a, b) to label the vacuole. The signal from 3xYpet-RBB1 (c) for the complemented plant is shown. (d-f) 3xYpet-RBB1 does not co-localize with FM4-64. Images show the cotyledon of 7-d-old seedlings expressing 3xYpet-RBB1 (d) stained with 5 μM FM4-64 (e) and the merged image (f). (g-i) 3xYpet-RBB1 and RFP-SYP22 do not co-localize. Cotyledons of 7-d-old seedlings expressing 3xYpet-RBB1 (g) and RFP-SYP22 (h) were imaged. Merged image (i) is shown. (j-l) 3xYpet-RBB1 and Lysotracker Red do not co-localize in rosette leaves. Seedlings from the 3xYpet-RBB1 line were stained with Lysotracker Red. Signal from 3xYpet-RBB1 (j), Lysotracker Red (k) and the merged image (l) are shown. Arrowheads indicate the localization of 3xYpet-RBB1 at the tip of elongating trans-vacuolar strands. (m-o) GFP molecules can also label the tips of transvacuolar strands. Seedlings from a 35::GFP marker line were stained with Lysotracker Red. The GFP signal (m), Lysotracker Red (n) and the merged image (o) are shown. Arrowheads indicate the tip of TVS. All scale bars = 20 μm. (p) 3xYpet-RBB1 accumulates in the soluble (S100) and membrane pellet (P100) fractions from whole seedlings. Immunoblot of 3xYpet-RBB1 soluble (S100) and membrane (P100) fractions from Col-0 and 3xYpet-RBB1 transgenic plants using antibodies against GFP (α-GFP), cFBPase (α-cFBPase, control for soluble fraction), and Plasma Membrane H+ATPase (α-H+ATPase, control for membrane fraction).

In order to characterize the function of *RBB1*, a fluorescent protein (FP) fusion was generated to visualize its protein localization. Given the large size of the *RBB1* coding sequence, we used Recombineering [32] to generate a FP fusion with RBB1 driven by its own promoter and other regulatory sequences. This system utilizes homologous recombination with TAC clones to fuse the gene encoding 3xYpet (YFP for energy transfer), a bright variation of YFP [48], to the gene of interest within the native genomic context. Both N- and C-terminal fusions of RBB1 with 3xYpet were generated and introduced into Col-0 wild type. We could not detect fluorescence in RBB1-3xYpet lines after selection of 15 independent transformants. However, 3xYpet-RBB1 was detected in cotyledon epidermal cells, root cortical cells and, although very faint, in the cortex of hypocotyls of light-grown seedlings (S5A and S5B Fig). No Ypet signal was detected at the root tip corresponding to the cell division zone. In order to determine if the 3xYpet-RBB1 fusion is functional, we introgressed the transgene into the *rbb1-2* allele by crossing. This experiment was carried out in *rbb1-2* because the GFP-TIP2;1 marker in *rbb1-1* is tightly linked to the *RBB1* locus and the probability of identifying *rbb1-1* segregants without GFP-TIP2;1 was very low. In contrast to *rbb1-2* seedlings stained with Lysotracker red, which showed many bulbs in cotyledons (Fig 6A), *rbb1-2* seedlings expressing the 3xYpet-RBB1 protein have vacuole morphology very similar to the wild type (Fig 6B and 6C). Therefore, the 3xYpet-RBB1 construct complements the bulb phenotype of *rbb1-2* indicating that this fusion protein is functional.

The localization of 3xYpet-RBB1 was diffuse and suggestive of cytosolic localization in cotyledons of 7-day-old seedlings (Fig 6D). In order to determine its sub-cellular distribution in detail, we tested for co-localization between 3xYpet-RBB1 and the plasma membrane dye FM4-64, a marker for the tonoplast, RFP-SYP22 [30], and the vacuole dye Lysotracker Red. The 3xYpet-RBB1 marker does not co-localize with FM4-64 (Fig 6D–6F) or with RFP-SYP22 (Fig 6G–6I). The marker was found in the cytosol and did not co-localize with Lysotracker Red (Fig 6J–6L), even though occasionally there was good co-localization between these two signals (S5C–S5E Fig). These results indicate 3xYpet-RBB1 protein is most likely a soluble protein that accumulates in the cytosol. We also detected 3xYpet-RBB1 in a punctate pattern inside the vacuole that seemed to correspond to the tip of TVS labeled with Lysotracker Red in confocal images (Fig 6J–6L). Interestingly, 3xYpet-RBB1 remained associated with the tip during the elongation of those TVS (S5F Fig) in a similar manner as actin bundles [49]. These TVS structures may represent cross-sections of membrane sheets that transverse the vacuole, as previously proposed [10, 49]. In order to determine the significance of this localization, we compared it to the localization of a soluble GFP marker in the same manner. As shown in Fig 6M–6O, a simple GFP marker can also label the tip of TVS, and therefore, the punctate localization of RBB1 may simply result from accumulation of cytoplasmic proteins at the tip of emerging TVS. Immunoblot analysis was used to confirm that the 3xYpet-RBB1 fusion protein accumulates as the expected size of ~400 kDa in seedlings. Membrane and soluble fractions were isolated from wild type Col-0 and the 3xYpet-RBB1 line and the fusion protein was detected with an anti-GFP antibody. The cytosolic fructose-1,6-bisphosphatase (cFBPase) and plasma membrane H^+^ATPase were used as soluble and membrane protein controls, respectively. 3xYpet-RBB1 was detected in both the soluble (S100) and the membrane (P100) fractions in 4 days-old seedlings (Fig 6L and S6 Fig). These results overall indicate that 3xYpet-RBB1 is a large soluble protein that is likely to associate with cellular membranes.

## Discussion

We have identified *rbb1-1*, a novel mutant with defects in vacuole morphology. Vacuoles in *rbb1-1* contain increased numbers of bulbs in the large central vacuole of cotyledons and hypocotyl cells when compared to the parental control. These defined vacuolar sub-structures can be visualized with fluorescent protein markers for the tonoplast [15, 23]. It was recently proposed that bulbs, specially when they have more than double the intensity of fluorescence when compared to the tonoplast, are artifacts that result from GFP dimerization that stabilizes the adhesion of tonoplast membranes [24]. This model, however, does not explain the accumulation of bulbs in the 2xFYVE-YFP line in which the fusion protein associates with the membrane in a reversible manner by binding to a specific phosphoinositide [15, 23, 50]. The GFP-TIP2;1-labeled bulbs in *rbb1-1* showed between 2 and 3.5-fold higher fluorescence intensity when compared to the tonoplast, indicating that they are likely to have a similar structure as previously characterized IVPS and bulbs [15, 23, 24]. We can not discard the possibility that the GFP fusion enhances the stability of the bulbs in *rbb1-1*, but *rbb1* mutant alleles comprise a sensitized genetic background for studying the formation and regulation of these structures, which are quite elusive under normal conditions. The two other mutants that have been identified as having abnormal bulb accumulation, *sgr2* and *vti11*, have fewer bulbs, also have very abnormal vacuole morphology [23, 25, 28]. Hence, *rbb1-1* is a unique tool to characterize the structure and function of the bulbs in the absence of other vacuolar defects.

*RBB1* encodes a very large protein with unknown molecular function. In order to determine the function of *RBB1*, we generated a 3xYpet-RBB1 fusion protein under the control of the *RBB1* promoter. This construct complemented the *rbb1-2* mutant phenotype indicating that the 3xYpet-RBB1 fusion is functional. In seedlings, 3xYpet-RBB1 was detected in the root in the elongation and differentiation zones, but not in the cell division zone, and in hypocotyl and cotyledon epidermal cells. Therefore, 3xYpet-RBB1 accumulates in cells that contain a large lytic vacuole [51], consistent with a role of RBB1 in regulating the morphology of this important organelle. Confocal scanning laser microscopy with the 3xYpet-RBB1 line indicated that this protein accumulates in the cytosol, and that in a few instances also co-localizes with Lysotracker red, a fluorescent dye that labels the tonoplast. RBB1 was found both in the soluble and membrane fractions by fractionation experiments, which raises the possibility that RBB1 may associate with membranes. 3xYpet-RBB1 was often present at the tips of elongating TVS in cotyledons and leaves, but visualization of a GFP control indicated that this localization may be detected some times with other cytosolic proteins.

We detected a significant decrease in the number of TVS in *rbb1-1* mutants both in light- and dark-grown hypocotyls and in cotyledons of light-grown seedlings. The TVS phenotype of *rbb1-1* supports the hypothesis that RBB1 functions in the formation or stability of TVS. This hypothesis is supported by the observation that both the bulb phenotype and the reduced TVS phenotype can be detected at day 3 during germination. TVS are very dynamic structures that require the actomyosin cytoskeleton [4, 5, 14, 49], and are thought redistribute cytoplasmic contents or to enhance the structural stability of the cytoskeleton [14]. A potential role of RBB1 in TVS formation is consistent with the expression of 3xYpet-RBB1 in cells that contain a large vacuole. Given the localization of 3xYpet-RBB1 to the cytosol and its accumulation in both the soluble and membrane fractions, it is tempting to speculate that RBB1 interacts with the tonoplast during the formation and elongation of TVS and contributes to the remodeling of the vacuolar membrane. However, more experiments beyond the scope of this work are needed to test this possibility. Moreover, we speculate that the increased number of bulbs in *rbb1-1* results from its inability to form functional TVS. Therefore, we propose an alternative model for bulb biogenesis in which a bulb forms when a TVS initiates but fails to elongate and the bulb functions as a mean to accommodate tonoplast membranes and cytoskeletal proteins that may have accumulated at the initiation site, until the bulb membrane is reabsorbed back into the tonoplast. It follows that the bulbs may have no specific function in the vacuole other than the transient accumulation of membranes and proteins when TVS failed to elongate. Further characterization of the function of RBB1 and interacting partners may shed light in the mechanisms that remodel the vacuole membrane into TVS or bulbs. Unlike the effects of GFP dimerization in vacuole morphology after fusion with vacuolar H+-pyrophosphatase [24], it is unlikely that GFP dimerization plays a big role in the TVS phenotype of *rbb1*. While the bulb fluorescence intensity for cotyledons was 3.5 fold higher than that of tonoplast, this ratio is only 2.0 for hypocotyls in *rbb1-1*, the same tissue that was used to quantify TVS in Fig 2. Therefore, a ratio higher than 2.0 is not necessary in *rbb1-1* to induce the accumulation of bulbs or a reduction in TVS.

Previously proposed functions of vacuolar bulbs include acting as a tonoplast reservoir for rapid growth or serving as the sites of degradation of tonoplast membrane and proteins (Saito et al. 2002 and Maîtrejean et al. 2011). We tested one of these hypotheses by measuring the hypocotyl growth of *rbb1-1* in the dark, a condition that promotes cell elongation. However, no obvious growth differences were observed between *rbb1-1* and the parental line. One possibility is that the additional tonoplast membranes contained in *rbb1-1* bulbs do not contribute to cell elongation. Alternatively, other factors such as the elasticity of the cell wall [49] may be limiting and would prevent us from detecting a difference in this assay.

The *RBB1* gene is expressed in seedlings and adult plants both in shoots and roots. That *RBB1* is expressed as a full-length transcript of 8,957 bp is supported by several ESTs as well as the alignment of RNAseq reads spanning the entire coding sequence by the Integrated Genome Browser [52, 53]. The molecular function of RBB1 is unknown as the protein has no conserved domains, and it represents a new plant-specific protein. The identification of proteins that interact with RBB1 may shed light on the molecular mechanisms by which RBB1 could regulate the morphology of the vacuole.

## Supporting Information

S1 Fig *rbb1-1* mutants show normal growth. (a-b) Normal growth phenotype of 4 day-old seedlings (a), and 6 week-old plants (b) from the parental line, *rbb1-1*, Col-0, and *rbb1-2*. (c) Relative GFP content in the parental line and *rbb1-1* by immunoblotting. The intensity values for GFP from Fig 1O were normalized against the Calreticulin (CRT) loading control.
(TIF)

S2 Fig Maximum fluorescence intensity for bulbs and tonoplast. (a, b) Examples of fluorescence intensity profiles that were used to estimate intensity values in the parental line (a) and *rbb1-1* (b). Intensity value profiles from a line selection (white lines) across each bulb or the tonoplast were extracted in Image J from confocal images. Only the maximum value was collected for each line selection (arrowheads). (c, d) Maximum fluorescence intensity of bulbs and the tonoplast from cotyledon (c) and hypocotyl cells (d) in the parental line or *rbb1-1* grown in the light. Images were analyzed as in (a, b). Data shown is the average of maximum intensity values for 10–15 images from at least 3 seedlings each. The numbers inside the white bars correspond to the calculated ratio between the maximum fluorescence intensity in the bulbs and that of the tonoplast. Identical microscope settings were used when collecting data for cotyledons or when collecting data for hypocotyls.
(TIF)

S3 Fig Quantification of TVS and growth phenotypes of *rbb1-1*. (a-b) An example of images used for TVS quantification. Thick optical sections were captured from hypocotyls (a). A seedling from the parental line is shown. The boxed area is enlarged in (b) to show the TVS that were counted (arrowhead). Bar = 20 μm. (c-d) The parental line and *rbb1-1* have similar hypocotyl growth in the dark. Seedlings were grown in the dark for up to 5 days and seedlings were imaged in a scanner. Hypocotyl length was measured using Image J (NIH) from seedlings at days 3 and 5 (n = 10). Black bars represent parental line; gray bars represent *rbb1-1* mutant.
(TIF)

S4 Fig Lysotracker Red labels the tonoplast in addition to the vacuole lumen in Arabidopsis. GFP-TIP2;1 seedlings were stained with Lysotracker Red for 2 h and imaged by confocal microscopy. The tonoplast marker GFP-TIP2;1 (green, a) co-localizes with the membrane signal of Lysotracker Red (magenta, b). White signal in the merged image (c) represents co-localized pixels. Scale bar = 20 μm.
(TIF)

S5 Fig Localization of 3xYpet-RBB1. (a-b) 3xYpet-RBB1 can be detected in cotyledons, hypocotyls and roots. Four-day-old seedlings expressing 3xYpet-RBB1 under the control of its native promoter were imaged by confocal microscopy. An overlay of the fluorescence signal and the bright field image is shown in (a) and the fluorescence signal is shown in (b). The arrow in (b) indicates the position of the root tip. Scale bar = 200μm. (c-e) An example where the 3xYpet-RBB1 co-localizes with Lysotracker Red. 3xYpet-RBB1 is shown in green (c), Lysotracker Red is shown in magenta (d) and the merged image is shown (e). Scale bar = 10 μm. (f) The 3xYpet-RBB1 signal remains associated with the TVS tip. Cotyledons that were stained with Lysotracker were captured by time lapse microscopy every 7.75 sec. Note that the 3xYpet-RBB1 signal (green) remains associated with the tip of the elongating TVS labeled with Lysotracker Red (magenta). Scale bar = 10 μm.
(TIF)

S6 Fig 3xYpet-RBB1 Immunoblot. This blot corresponds to the image shown in Fig 6O but includes the entire blot to show the size of the 3xYpet-RBB1 fusion. * Non-specific bands.
(TIF)

S1 Movie Z-stack of Col-WT cotyledon stained with Lysotracker Red. Four-day-old Col-WT seedlings stained with Lysotracker Red as in Fig 5F. The stack represents 20 optical sections every 1 μm. Image dimensions are x:106.17 μm, y:106.17 μm.
(AVI)

S2 Movie Z-stack of *rbb1-2* cotyledon stained with Lysotracker Red. Four-day-old *rbb1-2* seedlings stained with Lysotracker Red as in Fig 5G. The stack represents 45 optical sections every 1 μm. Image dimensions are x:106.17 μm, y:106.17 μm.
(AVI)

S1 Table List of primers used for Recombineering. (DOCX)

S2 Table List of primers used for RT-PCR. (DOCX)

## References

[1] Bassham DC, Brandizzi F, Otegui MS, Sanderfoot AA. The secretory system of Arabidopsis. The Arabidopsis Book 2008. p. 1–29. 10.1199/tab.0116 PMC3243370 22303241

[2] Muntz K. Protein dynamics and proteolysis in plant vacuoles. J Exp Bot. 2007;58(10):2391–407. 10.1093/jxb/erm089 17545219

[3] Xiang L, Etxeberria E, Van den Ende W. Vacuolar protein sorting mechanisms in plants. FEBS J. 2013;280(4):979–93. 10.1111/febs.12092 23241209

[4] Ruthardt N, Gulde N, Spiegel H, Fischer R, Emans N. Four-dimensional imaging of transvacuolar strand dynamics in tobacco BY-2 cells. Protoplasma. 2005;225(3–4):205–15. 10.1007/s00709-005-0093-7 16228899

[5] Uemura T, Yoshimura SH, Takeyasu K, Sato MH. Vacuolar membrane dynamics revealed by GFP-AtVam3 fusion protein. Genes to Cells. 2002;7(7):743–53. 12081650 10.1046/j.1365-2443.2002.00550.x

[6] Nebenfuhr A, Gallagher LA, Dunahay TG, Frohlick JA, Mazurkiewicz AM, Meehl JB, et al Stop-and-go movements of plant Golgi stacks are mediated by the acto-myosin system. Plant Physiol. 1999;121(4):1127–42. 10594100 10.1104/pp.121.4.1127 PMC59480

[7] Tominaga M, Yokota E, Vidali L, Sonobe S, Hepler PK, Shimmen T. The role of plant villin in the organization of the actin cytoskeleton, cytoplasmic streaming and the architecture of the transvacuolar strand in root hair cells of Hydrocharis. Planta. 2000;210(5):836–43. 10.1007/s004250050687 10805457

[8] Tominaga M, Sonobe S, Shimmen T. Mechanism of Inhibition of Cytoplasmic Streaming by Auxin in Root Hair Cells of Hydrocharis. Plant Cell Physiol. 1998;39(12):1342–9.

[9] Kutsuna N, Hasezawa S. Dynamic Organization of Vacuolar and Microtubule Structures during Cell Cycle Progression in Synchronized Tobacco BY-2 Cells. Plant Cell Physiol. 2002;43(9):965–73. 10.1093/pcp/pcf138 12354913

[10] Higaki T, Kutsuna N, Okubo E, Sano T, Hasezawa S. Actin Microfilaments Regulate Vacuolar Structures and Dynamics: Dual Observation of Actin Microfilaments and Vacuolar Membrane in Living Tobacco BY-2 Cells. Plant Cell Physiol. 2006;47(7):839–52. 10.1093/pcp/pcj056 16672254

[11] Staiger CJ, Yuan M, Valenta R, Shaw PJ, Warn RM, Lloyd CW. Microinjected profilin affects cytoplasmic streaming in plant cells by rapidly depolymerizing actin microfilaments. Curr Biol. 1994;4(3):215–9. doi: S0960-9822(00)00050-6 7922326 10.1016/s0960-9822(00)00050-6

[12] Yokota E, Vidali L, Tominaga M, Tahara H, Orii H, Morizane Y, et al Plant 115-kDa Actin-Filament Bundling Protein, P-115-ABP, is a Homologue of Plant Villin and is Widely Distributed in Cells. Plant Cell Physiol. 2003;44(10):1088–99. 10.1093/pcp/pcg132 14581634

[13] van der Honing HS, de Ruijter NC, Emons AM, Ketelaar T. Actin and myosin regulate cytoplasm stiffness in plant cells: a study using optical tweezers. New Phytol. 2010;185(1):90–102. 10.1111/j.1469-8137.2009.03017.x 19761443

[14] Hoffmann A, Nebenführ A. Dynamic rearrangements of transvacuolar strands in BY-2 cells imply a role of myosin in remodeling the plant actin cytoskeleton. Protoplasma. 2004;224(3):201–10. 10.1007/s00709-004-0068-0 15614481

[15] Saito C, Ueda T, Abe H, Wada Y, Kuroiwa T, Hisada A, et al A complex and mobile structure forms a distinct subregion within the continuous vacuolar membrane in young cotyledons of Arabidopsis. Plant J. 2002;29(3):245–55. 11844103 10.1046/j.0960-7412.2001.01189.x

[16] Hunter PR, Craddock CP, Di Benedetto S, Roberts LM, Frigerio L. Fluorescent reporter proteins for the tonoplast and the vacuolar lumen identify a single vacuolar compartment in Arabidopsis cells. Plant Physiol. 2007;145(4):1371–82. 17905861 10.1104/pp.107.103945 PMC2151705

[17] Bottanelli F, Foresti O, Hanton S, Denecke J. Vacuolar transport in tobacco leaf epidermis cells involves a single route for soluble cargo and multiple routes for membrane cargo. Plant Cell. 2011. 10.1105/tpc.111.085480 PMC3180807 21856792

[18] Escobar NM, Haupt S, Thow G, Boevink P, Chapman S, Oparka K. High-throughput viral expression of cDNA-green fluorescent protein fusions reveals novel subcellular addresses and identifies unique proteins that interact with plasmodesmata. Plant Cell. 2003;15(7):1507–23. 12837943 10.1105/tpc.013284 PMC165397

[19] Maitrejean M, Vitale A. How are tonoplast proteins degraded? Plant Signal Behav. 2011;6(11):1809–12. doi: 17867 [pii] 10.4161/psb.6.11.17867 22057339 PMC3329355

[20] Maitrejean M, Wudick MM, Voelker C, Prinsi B, Mueller-Roeber B, Czempinski K, et al Assembly and sorting of the tonoplast potassium channel AtTPK1 and its turnover by internalization into the vacuole. Plant Physiol. 2011;156(4):1783–96. 10.1104/pp.111.177816 21697507 PMC3149923

[21] Beebo A, Thomas D, Der C, Sanchez L, Leborgne-Castel N, Marty F, et al Life with and without *AtTIP1;1*, an Arabidopsis aquaporin preferentially localized in the apposing tonoplasts of adjacent vacuoles. Plant Mol Biol. 2009;70(1–2):193–209. 10.1007/s11103-009-9465-2 19229639

[22] Hicks GR, Rojo E, Hong S, Carter DG, Raikhel NV. Geminating pollen has tubular vacuoles, displays highly dynamic vacuole biogenesis, and requires VACUOLESS1 for proper function. Plant Physiol. 2004;134(3):1227–39. 14988481 10.1104/pp.103.037382 PMC389947

[23] Saito C, Uemura T, Awai C, Tominaga M, Ebine K, Ito J, et al The occurrence of 'bulbs', a complex configuration of the vacuolar membrane, is affected by mutations of vacuolar SNARE and phospholipase in Arabidopsis. Plant J. 2011;68(1):64–73. 10.1111/j.1365-313X.2011.04665.x 21645145

[24] Segami S, Makino S, Miyake A, Asaoka M, Maeshima M. Dynamics of Vacuoles and H+-Pyrophosphatase Visualized by Monomeric Green Fluorescent Protein in Arabidopsis: Artifactual Bulbs and Native Intravacuolar Spherical Structures. Plant Cell. 2014;26(8):3416–34. 10.1105/tpc.114.127571 25118245 PMC4371836

[25] Kato T, Morita MT, Fukaki H, Yamauchi Y, Uehara M, Niihama M, et al SGR2, a phospholipase-like protein, and zig/sgr4, a SNARE, are involved in the shoot gravitropism of Arabidopsis. Plant Cell. 2002;14(1):33–46. 11826297 10.1105/tpc.010215 PMC150549

[26] Ebine K, Okatani Y, Uemura T, Goh T, Shoda K, Niihama M, et al A SNARE complex unique to seed plants is required for protein storage vacuole biogenesis and seed development of Arabidopsis thaliana. Plant Cell. 2008;20(11):3006–21. 10.1105/tpc.107.057711 18984676 PMC2613668

[27] Sanderfoot AA, Kovaleva V, Bassham DC, Raikhel NV. Interactions between syntaxins identify at least five SNARE complexes within the Golgi/prevacuolar system of the Arabidopsis cell. Mol Biol Cell. 2001;12(12):3733–43. 11739776 10.1091/mbc.12.12.3733 PMC60751

[28] Zheng J, Han SW, Rodriguez-Welsh MF, Rojas-Pierce M. Homotypic Vacuole Fusion Requires VTI11 and Is Regulated by Phosphoinositides. Mol Plant. 2014;7(6):1026–40. 10.1093/mp/ssu019 24569132

[29] Rivera-Serrano EE, Rodriguez-Welsh MF, Hicks GR, Rojas-Pierce M. A small molecule inhibitor partitions two distinct pathways for trafficking of tonoplast intrinsic proteins in Arabidopsis. PLoS ONE. 2012;7(9):e44735 10.1371/journal.pone.0044735PONE-D-12-19870 [pii]. 22957103 PMC3434187

[30] Geldner N, Denervaud-Tendon V, Hyman DL, Mayer U, Stierhof YD, Chory J. Rapid, combinatorial analysis of membrane compartments in intact plants with a multicolor marker set. Plant J. 2009;59(1):169–78. doi: TPJ3851 [pii] 10.1111/j.1365-313X.2009.03851.x 19309456 PMC4854200

[31] Grefen C, Donald N, Schumacher K, Blatt MR. A Ubiquitin-10 promoter-based vector set for fluorescent protein tagging facilitates temporal stability and native protein distribution in transient and stable expression studies. Plant J. 2010;64(2):355–65. 10.1111/j.1365-313X.2010.04322.x 20735773

[32] Zhou R, Benavente LM, Stepanova AN, Alonso JM. A recombineering-based gene tagging system for Arabidopsis. Plant J. 2011;66(4):712–23. 10.1111/j.1365-313X.2011.04524.x 21294796

[33] Clough SJ, Bent AF. Floral dip: a simplified method for *Agrobacterium*-mediated transformation of Arabidopsis thaliana. Plant J. 1998;16(6):735–46. 10069079 10.1046/j.1365-313x.1998.00343.x

[34] Lukowitz W, Gillmor CS, Scheible W-R. Positional cloning in Arabidopsis Why it feels good to have a Genome Initiative working for you. Plant Physiol. 2000;123(3):795–805. 10889228 10.1104/pp.123.3.795 PMC1539260

[35] Pagny S, Cabanes-Macheteau M, Gillikin JW, Leborgne-Castel N, Lerouge P, Boston RS, et al Protein recycling from the Golgi apparatus to the endoplasmic reticulum in plants and its minor contribution to calreticulin retention. Plant Cell. 2000;12(5):739–56. 10810147 10.1105/tpc.12.5.739 PMC139924

[36] Cutler SR, Ehrhardt DW, Griffitts JS, Somerville CR. Random GFP: cDNA fusions enable visualization of subcellular structures in cells of Arabidopsis at a high frequency. Proc Natl Acad Sci U S A. 2000;97(7):3718–23. 10737809 10.1073/pnas.97.7.3718 PMC16306

[37] Gattolin S, Sorieul M, Hunter PR, Khonsari RH, Frigerio L. In vivo imaging of the tonoplast intrinsic protein family in Arabidopsis roots. BMC Plant Biol. 2009;9:133. doi: 1471-2229-9-133 [pii] 10.1186/1471-2229-9-133 19922653 PMC2784467

[38] Alonso JM, Stepanova AN, Leisse TJ, Kim CJ, Chen H, Shinn P, et al Genome-wide insertional mutagenesis of Arabidopsis thaliana. Science. 2003;301(5633):653–7. 10.1126/science.1086391301/5633/653 [pii]. 12893945

[39] Daxinger L, Hunter B, Sheikh M, Jauvion V, Gasciolli V, Vaucheret H, et al Unexpected silencing effects from T-DNA tags in Arabidopsis. Trends Plant Sci. 2008;13(1):4–6. 10.1016/j.tplants.2007.10.007 18178509

[40] Laxmi A, Pan J, Morsy M, Chen R. Light Plays an Essential Role in Intracellular Distribution of Auxin Efflux Carrier PIN2 in *Arabidopsis thaliana* . PLoS ONE. 2008;3(1):e1510 10.1371/journal.pone.0001510 18231596 PMC2200863

[41] Jaillais Y, Fobis-Loisy I, Miege C, Gaude T. Evidence for a sorting endosome in Arabidopsis root cells. Plant J. 2008;53(2):237–47. doi: TPJ3338 [pii] 10.1111/j.1365-313X.2007.03338.x 17999644

[42] Nováková P, Hirsch S, Feraru E, Tejos R, van Wijk R, Viaene T, et al SAC phosphoinositide phosphatases at the tonoplast mediate vacuolar function in Arabidopsis. Proceedings of the National Academy of Sciences. 2014;111(7):2818–23. 10.1073/pnas.1324264111 24550313 PMC3932866

[43] Marchler-Bauer A, Lu S, Anderson JB, Chitsaz F, Derbyshire MK, DeWeese-Scott C, et al CDD: a Conserved Domain Database for the functional annotation of proteins. Nucleic Acids Res. 2011;39(Database issue):D225–9. 10.1093/nar/gkq1189 21109532 PMC3013737

[44] Goodstein DM, Shu S, Howson R, Neupane R, Hayes RD, Fazo J, et al Phytozome: a comparative platform for green plant genomics. Nucleic Acids Res. 2012;40(Database issue):D1178–86. 10.1093/nar/gkr944 22110026 PMC3245001

[45] Winter D, Vinegar B, Nahal H, Ammar R, Wilson GV, Provart NJ. An “Electronic Fluorescent Pictograph” Browser for Exploring and Analyzing Large-Scale Biological Data Sets. PLoS ONE. 2007;2(8):e718 10.1371/journal.pone.0000718 17684564 PMC1934936

[46] Zeller G, Henz SR, Widmer CK, Sachsenberg T, Rätsch G, Weigel D, et al Stress-induced changes in the Arabidopsis thaliana transcriptome analyzed using whole-genome tiling arrays. Plant J. 2009;58(6):1068–82. 10.1111/j.1365-313X.2009.03835.x 19222804

[47] Goda H, Sasaki E, Akiyama K, Maruyama-Nakashita A, Nakabayashi K, Li W, et al The AtGenExpress hormone and chemical treatment data set: experimental design, data evaluation, model data analysis and data access. Plant J. 2008;55(3):526–42. 10.1111/j.0960-7412.2008.03510.x 18419781

[48] Shaner NC, Steinbach PA, Tsien RY. A guide to choosing fluorescent proteins. Nat Methods. 2005;2(12):905–9. 10.1038/nmeth819 16299475

[49] Szymanski DB, Cosgrove DJ. Dynamic coordination of cytoskeletal and cell wall systems during plant cell morphogenesis. Curr Biol. 2009;19(17):R800–11. 10.1016/j.cub.2009.07.056 19906582

[50] Vermeer JE, van Leeuwen W, Tobena-Santamaria R, Laxalt AM, Jones DR, Divecha N, et al Visualization of PtdIns3P dynamics in living plant cells. Plant J. 2006;47(5):687–700. doi: TPJ2830 [pii] 10.1111/j.1365-313X.2006.02830.x 16856980

[51] Zouhar J, Rojo E. Plant vacuoles: where did they come from and where are they heading? Curr Opin Plant Biol. 2009;12(6):677–84. 10.1016/j.pbi.2009.08.004 19783468

[52] Gulledge AA, Roberts AD, Vora H, Patel K, Loraine AE. Mining Arabidopsis thaliana RNA-seq data with Integrated Genome Browser reveals stress-induced alternative splicing of the putative splicing regulator SR45a. Am J Bot. 2012;99(2):219–31. 10.3732/ajb.1100355 22291167

[53] Nicol JW, Helt GA, Blanchard SG Jr., Raja A, Loraine AE. The Integrated Genome Browser: free software for distribution and exploration of genome-scale datasets. Bioinformatics. 2009;25(20):2730–1. 10.1093/bioinformatics/btp472 19654113 PMC2759552

