# Supplementary figures and images for: REGULATOR OF BULB BIOGENESIS1 (RBB1) Is Involved in Vacuole Bulb Formation in Arabidopsis
Source: PLoS One. 2015 Apr 27;10(4):e0125621. doi: 10.1371/journal.pone.0125621 (PMC4411111; doi:10.1371/journal.pone.0125621)

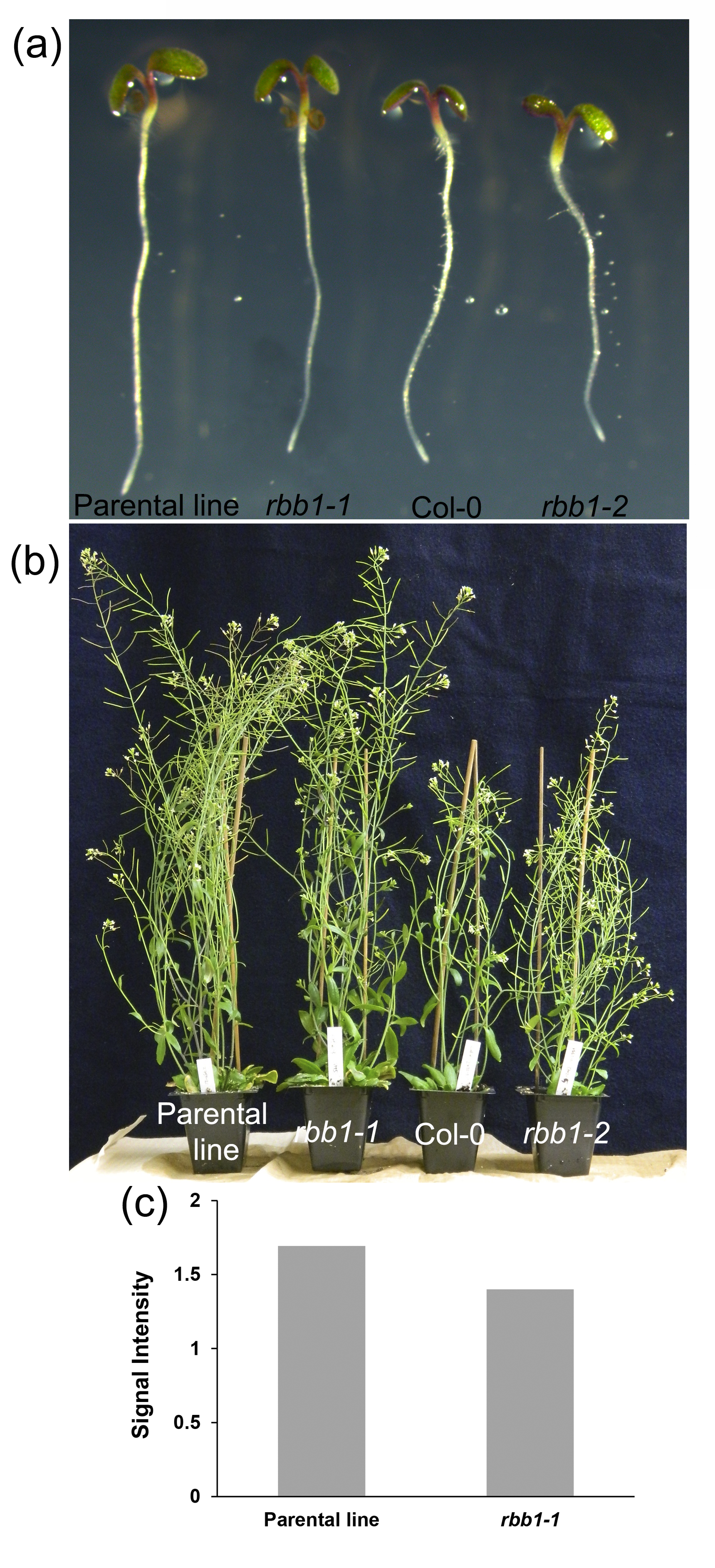

Supplement: S1 Fig — (a-b) Normal growth phenotype of 4 day-old seedlings (a), and 6 week-old plants (b) from the parental line, rbb1-1, Col-0, and rbb1-2. (c) Relative GFP content in the parental line and rbb1-1 by immunoblotting. The intensity values for GFP from Fig 1O were normalized against the Calreticulin (CRT) loading control. (TIF) [file pone.0125621.s001.tif]

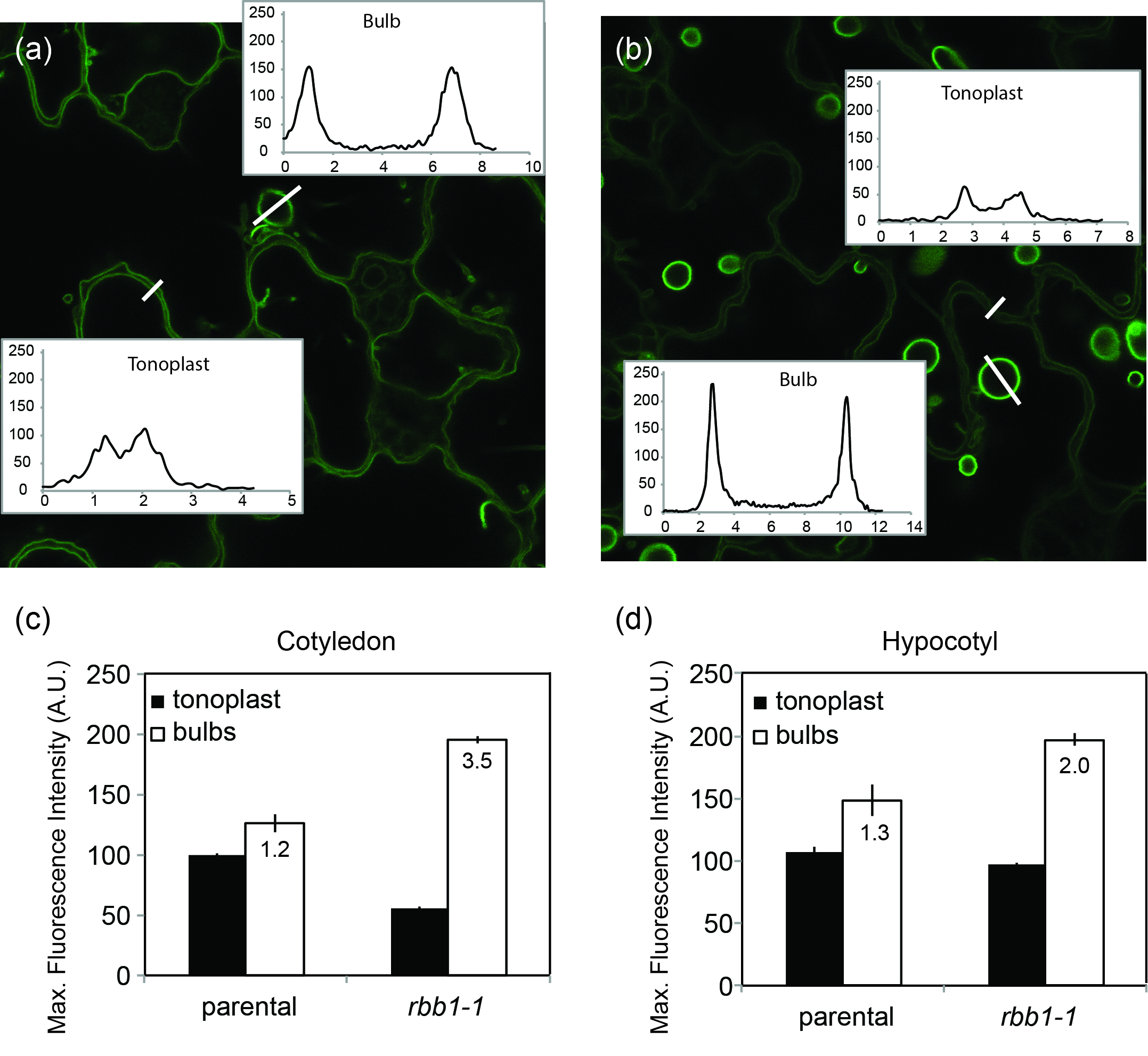

Supplement: S2 Fig — (a, b) Examples of fluorescence intensity profiles that were used to estimate intensity values in the parental line (a) and rbb1-1 (b). Intensity value profiles from a line selection (white lines) across each bulb or the tonoplast were extracted in Image J from confocal images. Only the maximum value was collected for each line selection (arrowheads). (c, d) Maximum fluorescence intensity of bulbs and the tonoplast from cotyledon (c) and hypocotyl cells (d) in the parental line or rbb1-1 grown in the light. Images were analyzed as in (a, b). Data shown is the average of maximum intensity values for 10–15 images from at least 3 seedlings each. The numbers inside the white bars correspond to the calculated ratio between the maximum fluorescence intensity in the bulbs and that of the tonoplast. Identical microscope settings were used when collecting data for cotyledons or when collecting data for hypocotyls. (TIF) [file pone.0125621.s002.tif]

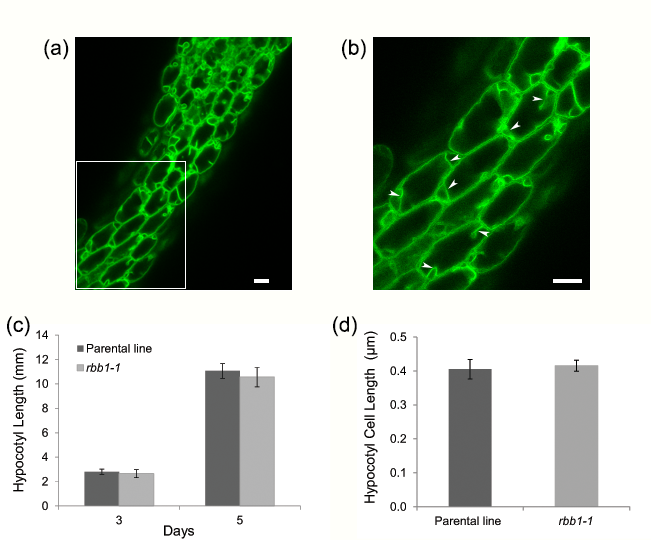

Supplement: S3 Fig — (a-b) An example of images used for TVS quantification. Thick optical sections were captured from hypocotyls (a). A seedling from the parental line is shown. The boxed area is enlarged in (b) to show the TVS that were counted (arrowhead). Bar = 20 μm. (c-d) The parental line and rbb1-1 have similar hypocotyl growth in the dark. Seedlings were grown in the dark for up to 5 days and seedlings were imaged in a scanner. Hypocotyl length was measured using Image J (NIH) from seedlings at days 3 and 5 (n = 10). Black bars represent parental line; gray bars represent rbb1-1 mutant. (TIF) [file pone.0125621.s003.tif]

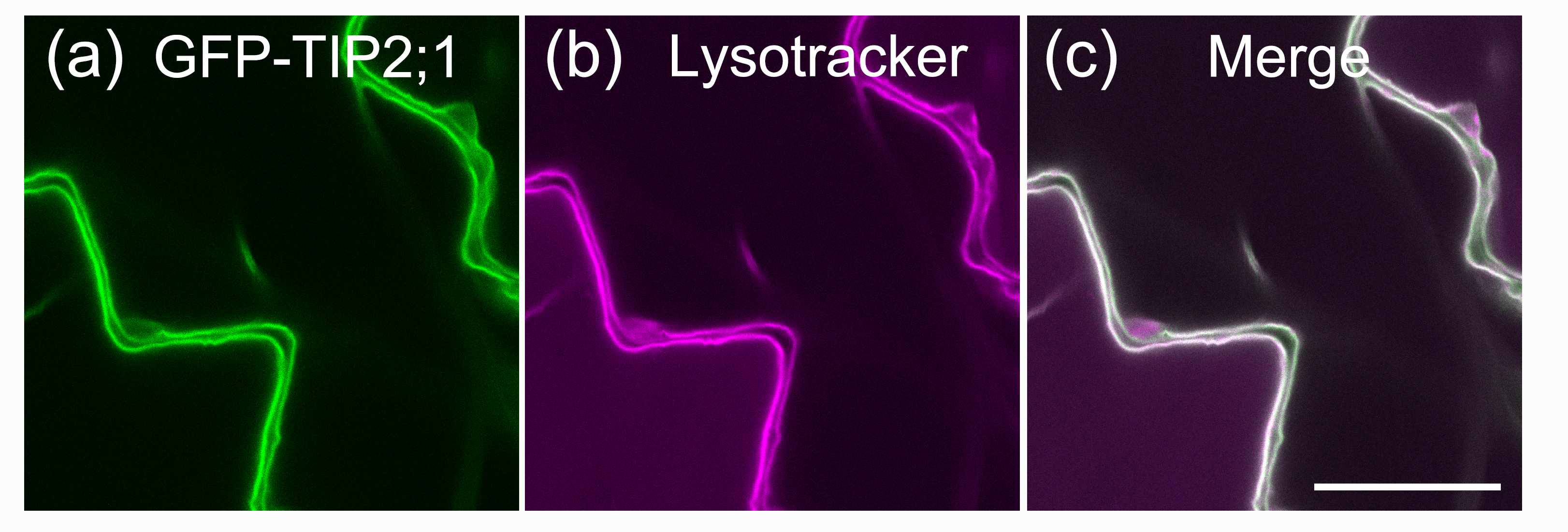

Supplement: S4 Fig — GFP-TIP2;1 seedlings were stained with Lysotracker Red for 2 h and imaged by confocal microscopy. The tonoplast marker GFP-TIP2;1 (green, a) co-localizes with the membrane signal of Lysotracker Red (magenta, b). White signal in the merged image (c) represents co-localized pixels. Scale bar = 20 μm. (TIF) [file pone.0125621.s004.tif]

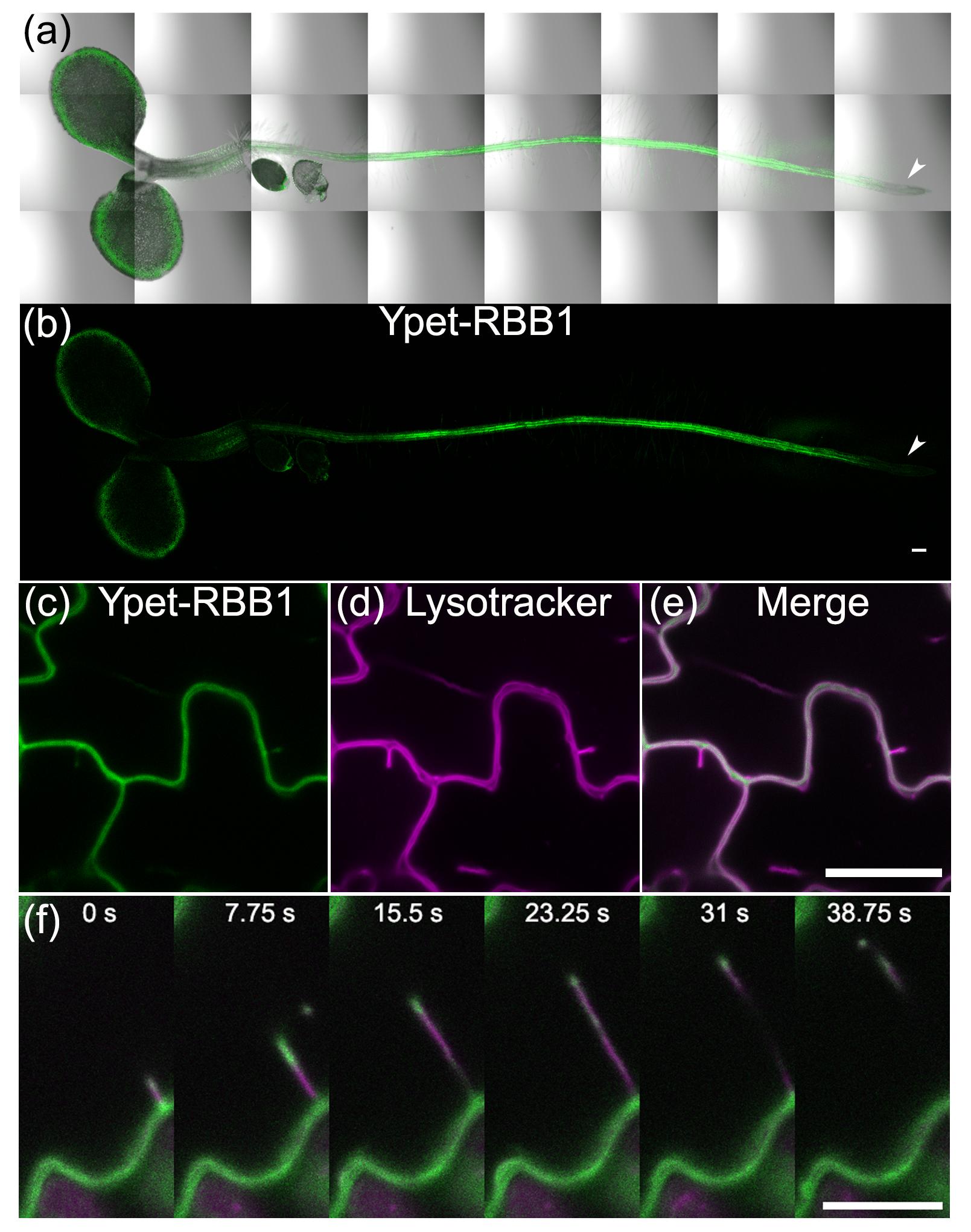

Supplement: S5 Fig — (a-b) 3xYpet-RBB1 can be detected in cotyledons, hypocotyls and roots. Four-day-old seedlings expressing 3xYpet-RBB1 under the control of its native promoter were imaged by confocal microscopy. An overlay of the fluorescence signal and the bright field image is shown in (a) and the fluorescence signal is shown in (b). The arrow in (b) indicates the position of the root tip. Scale bar = 200μm. (c-e) An example where the 3xYpet-RBB1 co-localizes with Lysotracker Red. 3xYpet-RBB1 is shown in green (c), Lysotracker Red is shown in magenta (d) and the merged image is shown (e). Scale bar = 10 μm. (f) The 3xYpet-RBB1 signal remains associated with the TVS tip. Cotyledons that were stained with Lysotracker were captured by time lapse microscopy every 7.75 sec. Note that the 3xYpet-RBB1 signal (green) remains associated with the tip of the elongating TVS labeled with Lysotracker Red (magenta). Scale bar = 10 μm. (TIF) [file pone.0125621.s005.tif]

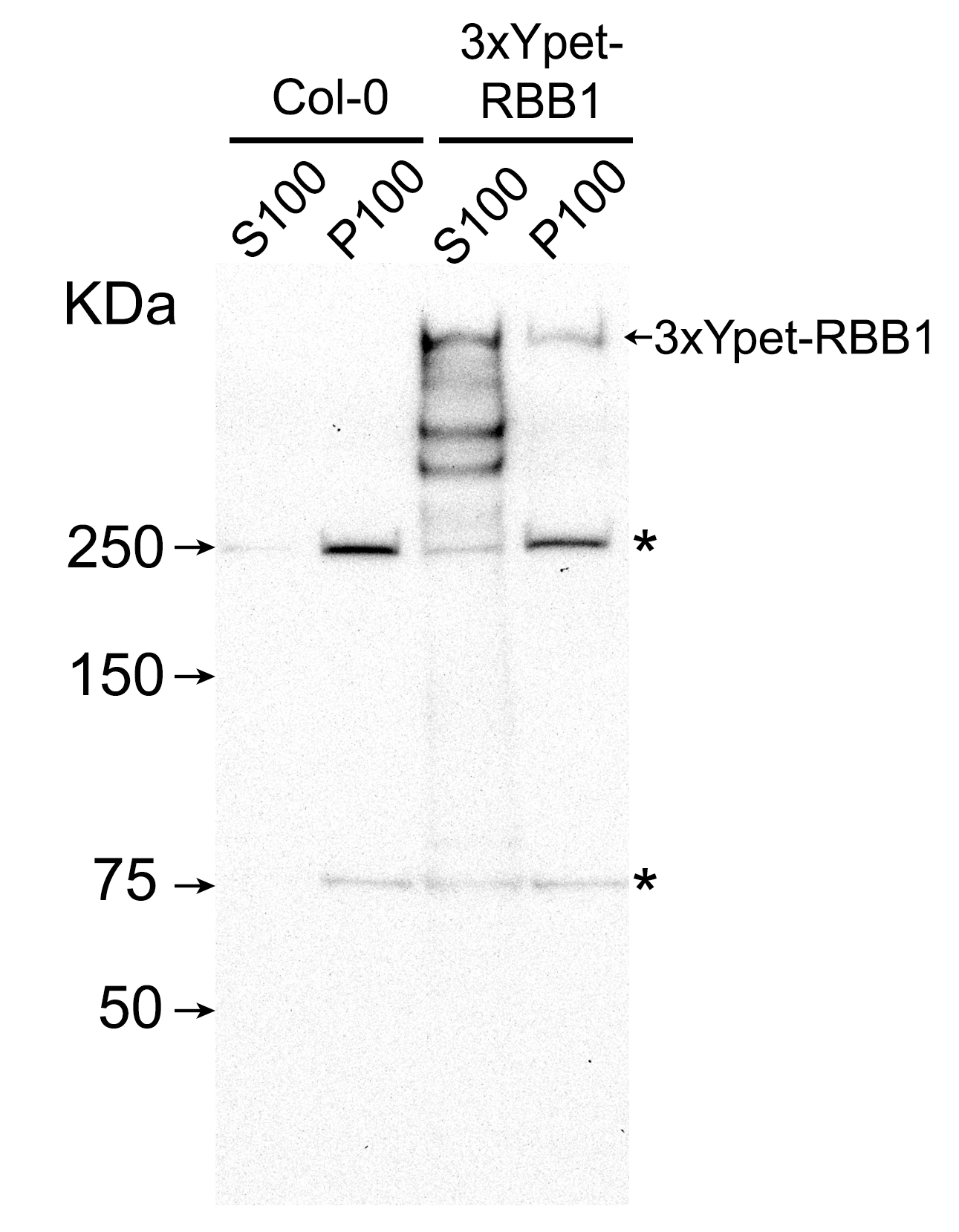

Supplement: S6 Fig — This blot corresponds to the image shown in Fig 6O but includes the entire blot to show the size of the 3xYpet-RBB1 fusion. * Non-specific bands. (TIF) [file pone.0125621.s006.tif]
