# Supplementary material for: REGULATOR OF BULB BIOGENESIS1 (RBB1) Is Involved in Vacuole Bulb Formation in Arabidopsis
Source: PLoS One. 2015 Apr 27;10(4):e0125621. doi: 10.1371/journal.pone.0125621 (PMC4411111; doi:10.1371/journal.pone.0125621)
Supplement: S1 Table — (DOCX) [file pone.0125621.s009.docx]

**S1 Table. List of primers used for Recombineering**

| **Name** | **Sequence** |
| --- | --- |
| GeneNF | ACTGTTCAAACCCTAAACCTCCAAATCATTTCTTCCCTGCATGAAAGATGGGAGGTGGAGGTGGAGCT |
| GeneNR | GATCATACCTTTGTAGAGACTGGTTCTTGGATGGTGACAACTCCAGTTTCGGCCCCAGCGGCCGCAGCAGCACC |
| GeneCF | AAGTACTCACTGGCAAATCTTCTCACACAACAAAGCCTTCATCACCTACAGGAGGTGGAGGTGGAGCT |
| GeneCR | TAAAAAACCTTTTATAGACACTGATTGTGTTTTTCTTGTTTCTTCACTTAGGCCCCAGCGGCCGCAGCAGCACC |
| DelLeft | AGGTAGTGTGGAGACATGAGCAGCTCAAATAGAGACAGCGAAAAGGAGGCttaccaatgcttaatcagtg |
| RB-amp | TATATTGCTCTAATAAATTTTTGGCGCGCCGGCCAATTAGGCCCGGGCGGttcaaatatgtatccgctcatg |
| NFtest | CAAAAAGGAAAGAAGGTAGAAACA |
| NRtest | CAAAAGGCCCACAAAAGATT |
| CFtest | AGTGCCGGAGAGAATTAGCC |
| CRtest | CCAAAAAGAGCCTAAAACAGAGG |
| DelLefttest | TAGATCGCAAGGAGGCAAGT |
