# Supplementary material for: REGULATOR OF BULB BIOGENESIS1 (RBB1) Is Involved in Vacuole Bulb Formation in Arabidopsis
Source: PLoS One. 2015 Apr 27;10(4):e0125621. doi: 10.1371/journal.pone.0125621 (PMC4411111; doi:10.1371/journal.pone.0125621)
Supplement: S2 Table — (DOCX) [file pone.0125621.s010.docx]

**S2 Table. List of primers used for RT-PCR**

| **Name** | **Sequence** |
| --- | --- |
| At5g40450_5’end-F | GGAAACTGGAGTTGTCACCA |
| At5g40450_5’end-R | CTGGCTTTCCGGTAGTATCC |
| At5g40450_mid-F | GAAGACAGATGCAGAACCGAG |
| At5g40450_mid-R | CTCCTCACTGCTTTCGCCTTG |
| At5g40450_3’end-F | CGGTCTTGCAGGGAAATCTC |
| At5g40450_3’end-R | TGCTTACCCTCCTCTTGCTC |
| UBQ5-F | GACGCTTCATCTCGTCC |
| UBQ5-R | CCACAGGTTGCGTTAG |
